# Supplementary material for: Synthesis and Biological Evaluation of a Caffeic Acid Phenethyl Ester Derivatives as Anti-Hepatocellular Carcinoma Agents via Inhibition of Mitochondrial Respiration and Disruption of Cellular Metabolism
Source: Cancers (Basel). 2025 Dec 27;18(1):92. doi: 10.3390/cancers18010092 (PMC12784929; doi:10.3390/cancers18010092)
Supplement: Supplementary file 1 [file cancers-18-00092-s001.zip › cancers-4045194-supplementary.pdf]

Supporting Information

Synthesis and Biological Evaluation of a Series of Caffeic acid phenethyl ester Derivatives as Anti-Hepatocellular Carcinoma Agents via Inhibition of Mitochondrial Respiration and Induction of Metal Ion-Associated Cell Death

Author＆Affiliation

Hao Dong1, Yuan Gao1#, Dongyue Jiang1#, Chenjie Feng1, Xinyue Gu2, Xiyunyi Cai3, Yulin Liu1, Guangyu Zhang4, Jiacheng Wen4, Weiwei Diao5, Qi Jiang1, Ying Zhou5, Ruixin Li1, Dayang Xu1, Weijia Xie4* and LiangWu1*

1 State Key Laboratory of Natural Medicines,China Pharmaceutical University, Nanjing 210009, People's Republic of China.

2 Department of Biology, The Johns Hopkins University, Baltimore, MD 21218, USA.

3 School of Life Science and Technology, China Pharmaceutical University, Nanjing 210009, People's Republic of China

4 Department of Medicinal Chemistry, School of Pharmacy, China Pharmaceutical University, Nanjing 210009, People's Republic of China

5 School of Traditional Chinese Pharmacy, China Pharmaceutical University, Nanjing 210009, People's Republic of China.

*Author to whom correspondence should be addressed.

#These authors contributed equally to this work.

Corresponding authors: （These authors contributed equally to this work.）

Liang Wu

Jiangsu Key Laboratory of Drug Screening, China Pharmaceutical University, Nanjing 210009, P.R. China. Institutional e-mail address: 1020132373@cpu.edu.cn; Tel.: +86 15651670600.

Weijia Xie

Department of Medicinal Chemistry, School of Pharmacy, China Pharmaceutical University, Nanjing 210009, People's Republic of China. Institutional E-mail address: weijiaxie@cpu.edu.cn; Tel.:+86 13813833567

Contents

Chemistry Methods and Data 4
Figure S1 Supplementary Data on the Validation of WX006's Tumor Growth Inhibitory Efficacy22
Figure S2 Supplementary Data on the Transcriptomic Effects of WX006 23
Figure S3 Supplementary Data on the Endoplasmic Reticulum Function Studies of WX006 23
Figure S4 Supplementary Data on the ferroptosis and cuproptosis Studies of WX006 24
Figure S5 Supplementary Data on the Mitochondrial Function Studies of WX006 25
Figure S6 Supplementary Data on the Inhibitory Effects of WX006 on the NDUFS2 Subunit 26
Figure S7 Supplementary Data on the In Vivo Tumor Growth Inhibition by WX006 27
Figure S8 Supplementary Data on the ^{1}H-NMR and ^{13}C-NMR Spectra of WX006 and other derivatives 28
Table S1 Detailed parameters used for molecular docking with DOCK6 56
Table S2 Detailed parameters used for molecular docking with Auto Dock Vina 56
Table S3 Primer sequences used in the qRT-PCR assay 57

Chemistry Methods and Data

Specific Synthesis route of compounds:

Synthesis route of 4,5-dibromobenzene-1,2-diol (22)

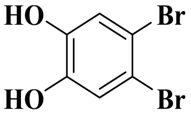

According to reference[78], compound **21** (30 g, 101 mmol) was dissolved in dichloromethane (DCM) in a two-necked flask. Under argon protection and at -78 °C, BBr_3 (56 g, 222 mmol) was added dropwise. The mixture was stirred for an additional 30 minutes at this temperature before warming to room temperature. After TLC monitoring indicated near-complete consumption of the starting material, water was added to quench the residual BBr_3. The mixture was extracted with DCM. The combined organic layers were washed with saturated sodium chloride solution, dried over anhydrous sodium sulfate, and concentrated under reduced pressure to afford a white solid, 4,5-dibromo-1,2-benzenediol **(22)**, which was used directly in the next step without further purification.

Synthesis route of 1,2-dibromo-4,5-bis((2-methoxyethoxy) methoxy) benzene (23)

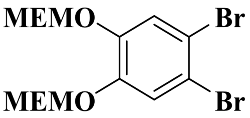

According to reference[79], 4,5-dibromo-1,2-benzenediol (**22**, 26.7 g, 100 mmol) was dissolved in acetone (10 mL). Anhydrous potassium carbonate (138 g, 1 mol) was added portionwise under ice-bath cooling. The mixture was stirred for 1 hour using a mechanical stirrer, followed by the slow addition of 1-chloromethoxy-2-methoxyethane (MEMCl, 25.2 g, 202 mmol). The reaction was then allowed to proceed at room temperature for 1 hour. After TLC confirmed the completion of the reaction, the mixture was filtered to remove K_2CO_3, and the acetone was removed under reduced pressure. The residue was allowed

to stand for 1 hour, during which the product solidified from a red-brown oily liquid into slightly pinkish white crystals **(23)**.

Synthesis route of diethyl 3,3'-(4,5-bis((2-methoxyethoxy) methoxy)-1,2-phenylene) (2*E*,2'*E*)-diacrylate (24)

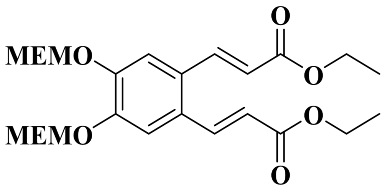

The above crystals (**23**, 20 g, 45 mmol), 1,3-bis(diphenylphosphino)propane (DPPP, 1.85 g, 4.5 mmol), and palladium (II) acetate ($Pd(OAc)_2$, 1 g, 4.5 mmol) were placed in a Schlenk tube and dispersed in 1,4-dioxane (100 mL). Triethylamine (18 g, 180 mmol) and ethyl acrylate (45 g, 450 mmol) were added sequentially. Under argon protection, the reaction was heated at 120 °C for 48h. After complete conversion, the system was cooled to room temperature and filtered to remove insoluble solids. The filtrate was diluted with ethyl acetate and washed sequentially with saturated sodium chloride solution, saturated sodium bicarbonate solution, and saturated ammonium chloride solution. The organic phase was dried over anhydrous sodium sulfate and concentrated under reduced pressure to give a red-brown oily liquid **(24)**, which was used directly in the next step.

Synthesis route of (2*E*,2'*E*)-3,3'-(4,5-bis((2-methoxyethoxy) methoxy)-1,2-phenylene) diacrylic acid (25)

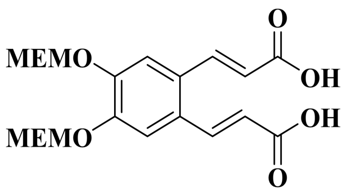

The oily liquid **(24)** was dissolved in ethanol (20 mL), and potassium hydroxide (7.7 g, 135 mmol) was added with vigorous stirring. After complete reaction, the mixture was diluted with water and acidified to pH 2 with 1N HCl under ice-bath cooling. The aqueous layer was extracted with ethyl acetate. The combined organic extracts were then washed with saturated sodium bicarbonate solution to transfer the carboxylic acid product into the aqueous phase as its carboxylate salt. The combined aqueous phases were re-acidified to pH 2 with 1N HCl under ice-bath cooling and extracted with ethyl acetate. The

combined organic layers were washed with saturated sodium chloride solution, dried over anhydrous sodium sulfate, and concentrated under reduced pressure to afford a brown-gray solid, compound **25**, with an overall yield of 90% over four steps.

General Procedure for the Synthesis of Compounds (26-37f):

According to reference[80], compound (25) (426 mg, 1 mmol) and the substituted aryl alcohol (2.2 mmol) were dissolved in dichloromethane (DCM, 2 mL). The coupling agent 1-ethyl-3-(3-dimethylaminopropyl) carbodiimide (EDCl, 1.53 g, 8 mmol) and 4-dimethylaminopyridine (DMAP, 268 mg, 2.2 mmol) were added. The reaction mixture was equipped with a nitrogen protection device and stirred at room temperature for 1 hour. After TLC monitoring confirmed the complete consumption of the starting material, the DCM was removed under reduced pressure. The residue was diluted with water and extracted with ethyl acetate. The combined organic layers were washed with saturated sodium chloride solution, dried over anhydrous sodium sulfate, and concentrated under reduced pressure. The crude product was purified by silica gel column chromatography (petroleum ether : ethyl acetate = 10 : 1) to afford the target compounds as brown oils in yields of 61-70%.

According to reference[81], the aforementioned compound (1 eq) was dissolved in methanol (1 mL), followed by the addition of 4N HCl (1 eq). The reaction mixture was refluxed at 70 °C for 2h. After near-complete consumption of the starting material, the reaction solution was directly concentrated under reduced pressure to afford a light yellow solid. The residue was extracted with ethyl acetate, and the organic layer was washed twice with saturated sodium chloride solution. The combined organic layers were dried over anhydrous sodium sulfate and concentrated under reduced pressure. The crude product was purified by silica gel column chromatography (petroleum ether : ethyl acetate = 4 : 1) to give the target compounds (**26-37f**) as pale yellow solids in yields of 62-68%.

General Procedure for the Synthesis of Compounds (38a-40c):

According to reference[82], substituted chiral 3-phenyl-2-propanol (2.2 mmol) and

triphenylphosphine (557 mg, 2.2 mmol) were dissolved in tetrahydrofuran (THF, 6 mL). Under ice-bath conditions, diisopropyl azodicarboxylate (DIAD, 444 mg, 2.2 mmol) was added, and the mixture was stirred for 15 minutes. Compound 25 (426 mg, 1 mmol) was dissolved in THF (2 mL), and the resulting solution was added dropwise to the reaction mixture. After TLC monitoring confirmed complete consumption of the starting material, water was added to quench the reaction. The mixture was extracted with ethyl acetate. The combined organic layers were washed with saturated sodium chloride solution, dried over anhydrous sodium sulfate, and concentrated under reduced pressure. The crude product was purified by silica gel column chromatography (petroleum ether : ethyl acetate = 6 : 1) to afford a white solid in 72-78% yield.

According to reference[81], the above compound (1 eq) was dissolved in methanol (1 mL), followed by the addition of 4N HCl (4 eq). The reaction mixture was refluxed at 70 °C for 2h. After near-complete consumption of the starting material, the reaction solution was directly concentrated under reduced pressure to give a light yellow solid. The residue was extracted with ethyl acetate, and the organic layer was washed twice with saturated sodium chloride solution. The combined organic layers were dried over anhydrous sodium sulfate and concentrated under reduced pressure. The crude product was purified by silica gel column chromatography (petroleum ether : ethyl acetate = 4 : 1) to yield the target compounds **(38a-40c**) as pale yellow solids in 61-68% yield.

Compound Data

diphenethyl 3,3'-(4,5-dihydroxy-1,2-phenylene)(2*E*,2'*E*)-diacrylate (26)(WX006)

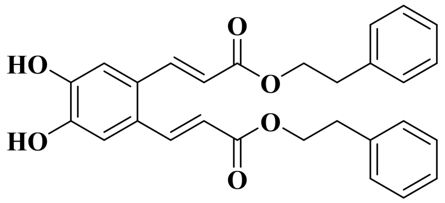

Slight yellow solid (40%); mp. 139-144℃; ^{1}H NMR (300 MHz, MeOD) δ 8.00 (d, *J* = 15.6 Hz, 2H), 7.33 – 7.19 (m, 10H), 7.10 (s, 2H), 6.24 (d, *J* = 15.6 Hz, 2H), 4.43 (t, *J* = 6.9 Hz, 4H), 3.03 (t, *J* = 6.9 Hz, 4H); ^{13}C NMR (75 MHz, MeOD) δ 167.11, 148.07, 140.69, 137.98, 128.65, 128.17, 126.56, 126.19, 117.44, 112.88, 65.03, 34.79; HRMS (ESI) m/z calcd for $C_{28}H_{26}O_6Na^+$ [M + Na] $^+$ 481.1622 found 481.1629.

bis(3-phenylpropyl) 3,3'-(4,5-dihydroxy-1,2-phenylene)(2*E*,2'*E*)-diacrylate (27)

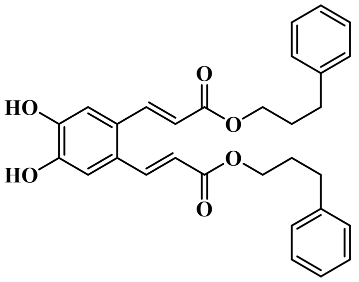

Slight yellow solid (36.5%); mp. 138-144℃; ^{1}H NMR (300 MHz, Acetone-*d*$_6$) δ 8.76 (s, 2H), 8.04 (d, *J* = 15.7 Hz, 2H), 7.31 – 7.15 (m, 12H), 6.30 (d, *J* = 15.6 Hz, 2H), 4.19 (t, *J* = 6.5 Hz, 4H), 2.75 (t, 4H), 2.04 – 1.96 (m, 4H); ^{13}C NMR (75 MHz, Acetone-*d*$_6$) δ 166.14, 147.79, 141.51, 140.30, 128.41, 128.35, 126.92, 125.85, 118.53, 113.51, 63.41, 31.91, 30.39; HRMS (ESI) m/z calcd for $C_{30}H_{30}O_6Na^+$ [M + Na] $^+$ 509.1935 found 509.1938.

bis(4-phenylbutyl) 3,3'-(4,5-dihydroxy-1,2-phenylene)(2*E*,2'*E*)-diacrylate (28)

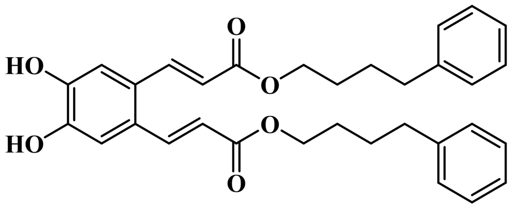

Slight yellow solid (37.7%); mp. 140-146℃; 1H NMR (300 MHz, Acetone-d_6) δ 8.80 (s, 2H), 8.03 (dd, J = 15.7, 1.6 Hz, 2H), 7.37 – 6.99 (m, 12H), 6.30 (d, J = 15.7 Hz, 2H), 4.23 (tt, J = 6.3, 2.7 Hz, 4H), 2.70 (tt, J = 7.2, 2.1 Hz, 4H), 1.93 – 1.52 (m, 8H); ^{13}C NMR (75 MHz, Acetone-d_6) δ 166.18, 147.79, 142.24, 140.24, 128.36, 128.25, 126.88, 125.68, 118.54, 113.47, 63.90, 35.16, 28.26, 27.78; HRMS (ESI) m/z calcd for $C_{32}H_{34}O_6Na^+$ [M + Na] $^+$ 537.2253 found 537.2257.

bis(4-methylphenethyl) 3,3'-(4,5-dihydroxy-1,2-phenylene)(2*E*,2'*E*)-diacrylate (29)

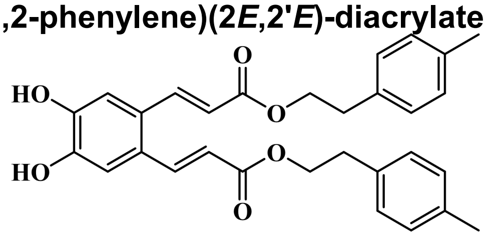

Slight yellow solid (38.2%); mp, 180-183℃; ^{1}H NMR (300 MHz, Acetone-d_6) δ 8.04 (d, *J* = 15.7 Hz, 2H), 7.27 – 7.14 (m, 10H), 6.30 (d, *J* = 15.6 Hz, 2H), 4.39 (t, *J* = 6.9 Hz, 4H), 2.99 (t, *J* = 6.9 Hz, 4H), 2.30 (s, 6H).^{13}C NMR (75 MHz, Acetone-d_6) δ 166.12, 147.76, 140.28, 135.73, 135.22, 129.10, 128.92, 126.90, 118.44, 113.41, 65.01, 34.55, 20.23; HRMS (ESI) m/z calcd for $C_{30}H_{30}O_6Na^+$ [M + Na] $^+$ 509.1935 found 509.1931.

bis(3-(p-tolyl)propyl) 3,3'-(4,5-dihydroxy-1,2-phenylene)(2*E*,2'*E*)-diacrylate (30)

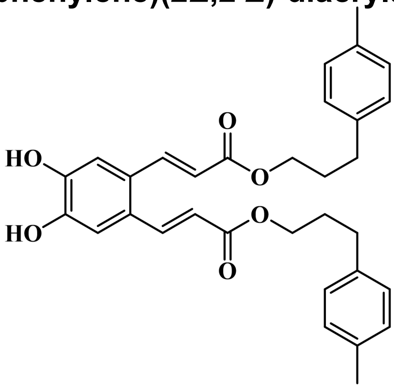

Slight yellow solid (37.6%); mp. 142-145℃; ^{1}H NMR (300 MHz, Methanol-d_4) δ 8.01 (d, *J* = 15.6 Hz, 2H), 7.12 (d, *J* = 8.6 Hz, 10H), 6.27 (d, *J* = 15.6 Hz, 2H), 4.21 (t, *J* = 6 Hz, 4H), 2.72 (t, *J* = 7.6 Hz, 4H), 2.29 (s, 6H), 2.03 (q, *J* = 7.0 Hz, 4H); ^{13}C NMR (75 MHz, Acetone-d_6) δ166.18, 147.75, 140.28, 138.41, 129.01, 128.35, 126.93, 118.54, 113.47,

63.47, 31.51, 30.49, 20.18; HRMS (ESI) m/z calcd for $C_{32}H_{34}O_6Na^+$ [M + Na] $^+$ 537.2253 found 537.2259.

bis(4-(p-tolyl)butyl) 3,3'-(4,5-dihydroxy-1,2-phenylene)(2*E*,2'*E*)-diacrylate (31)

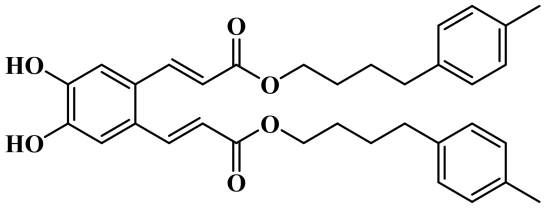

Slight yellow solid (36.8%); mp. 142-145℃; ^{1}H NMR (300 MHz, Acetone-d_6) δ 8.04 (d, J = 15.7 Hz, 1H), 7.27 (s, 1H), 7.13 (q, J = 5.7 Hz, 4H), 6.31 (d, J = 15.7 Hz, 1H), 4.23 (t, J = 5.8 Hz, 2H), 2.67 (t, J = 6.4 Hz, 2H), 2.29 (s, 3H), 1.73 – 1.69 (m, 4H); ^{13}C NMR (75 MHz, Acetone-d_6) δ 166.22, 140.23, 139.13, 134.89, 128.91, 128.31, 126.90, 118.55, 113.43, 63.96, 34.76, 27.90, 20.17; HRMS (ESI) m/z calcd for $C_{34}H_{38}O_6Na^+$ [M + Na] $^+$ 565.2561 found 565.2567.

bis(2-chlorophenethyl)3,3'-(4,5-dihydroxy-1,2-phenylene)(2*E*,2'*E*)-diacrylate (32a)

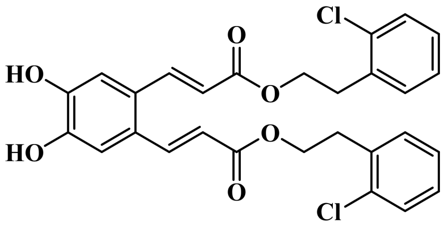

Slight yellow solid (39.6%); mp. 152-160℃; ^{1}H NMR (300 MHz, Acetone-d_6) δ 8.00 (d, J = 15.7 Hz, 2H), 7.43 – 7.23 (m, 10H), 6.26 (d, J = 15.6 Hz, 2H), 4.43 (t, J = 6.9 Hz, 4H), 3.17 (d, J = 7.1 Hz, 4H); ^{13}C NMR (75 MHz, Acetone-d_6) δ 166.09, 147.90, 140.42, 135.85, 133.80, 131.63, 129.46, 128.46, 127.30, 126.82, 118.21, 113.33, 63.13, 32.62; HRMS (ESI) m/z calcd for $C_{28}H_{24}O_6Cl_2Na^+$ [M + Na] $^+$ 549.0842 found 549.0845.

bis(3-chlorophenethyl)3,3'-(4,5-dihydroxy-1,2-phenylene)(2*E*,2'*E*)-diacryl ate (32b)

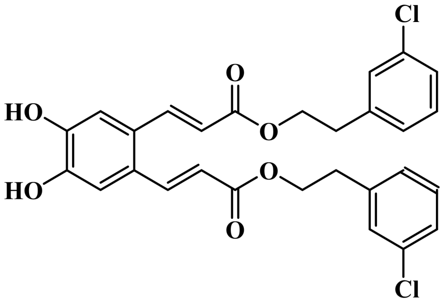

Slight yellow solid (44.1%); mp. 142-146℃; ^{1}H NMR (300 MHz, Acetone-d_6) δ 8.03 (d, J = 15.7 Hz, 2H), 7.41 –7.26 (m, 10H), 6.30 (d, J = 15.6 Hz, 2H), 4.44 (t, J = 6.8 Hz, 4H), 3.06 (t, J = 6.7 Hz, 4H); ^{13}C NMR (75 MHz, Acetone-d_6) δ 166.12, 147.90, 140.99, 140.45, 133.70, 130.13, 128.98, 127.72, 126.80, 126.55, 118.17, 113.35, 64.45, 34.51; HRMS (ESI) m/z calcd for $C_{28}H_{24}O_6Cl_2Na^+$ [M + Na] $^+$ 549.0842 found 549.0848.

bis(4-chlorophenethyl)3,3'-(4,5-dihydroxy-1,2-phenylene)(2*E*,2'*E*)-diacryl ate (32c)

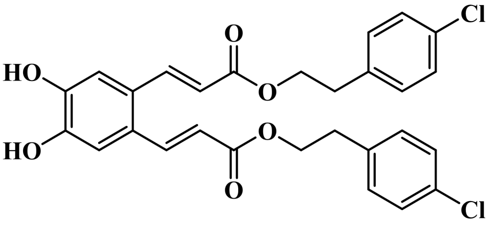

Slight yellow solid (41.5%); mp. 190-194℃; ^{1}H NMR (300 MHz, Acetone-d_6) δ 8.02 (d, J = 15.5 Hz, 2H), 7.39 – 7.36 (m, 8H), 7.26 (s, 2H), 6.29 (d, J = 15.6 Hz, 2H), 4.42 (t, J = 6.6 Hz, 4H), 3.05 (t, J = 6.5 Hz, 4H); ^{13}C NMR (75 MHz, Acetone-d_6) δ 166.12, 147.89, 140.37, 137.47, 131.79, 130.80, 128.46, 126.80, 118.21, 113.35, 64.58, 34.22; HRMS (ESI) m/z calcd for $C_{28}H_{24}O_6Cl_2Na^+$ [M + Na] $^+$ 549.0842 found 549.0844.

bis(3-(4-chlorophenyl)propyl)3,3'-(4,5-dihydroxy-1,2-phenylene)(2*E*,2'*E*)-diacrylate (33)

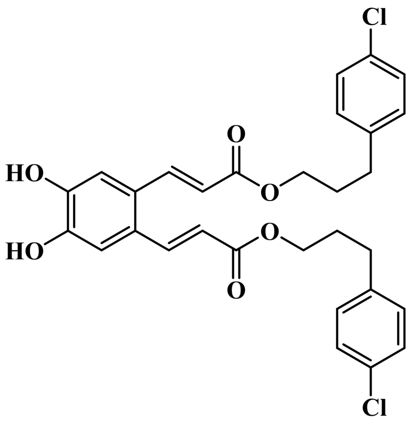

Slight yellow solid (39.3%); mp. 150-153℃； ^{1}H NMR (300 MHz, Acetone-d_6) δ 8.06 (d, *J* = 15.8 Hz, 2H), 7.35 – 7.28 (m, 10H), 6.34 (d, *J* = 15.9 Hz, 2H), 4.22 (t, 4H), 2.78 (t, *J* = 6.9 Hz, 4H), 2.07 – 1.91 (m, 4H); ^{13}C NMR (75 MHz, Acetone-d_6) δ166.21, 147.74, 140.50, 140.37, 131.19, 130.22, 128.39, 126.94, 118.45, 113.47, 63.33, 31.27, 30.27; HRMS (ESI) m/z calcd for $C_{30}H_{28}O_6Cl_2Na^+$ [M + Na] $^+$ 577.1155 found 577.1159.

bis(4-(4-chlorophenyl)butyl)3,3'-(4,5-dihydroxy-1,2-phenylene)(2*E*,2'*E*)-diacrylate (34)

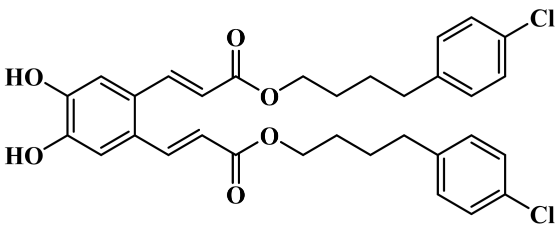

Slight yellow solid (38.2%); mp. 160-165℃; ^{1}H NMR (300 MHz, Acetone-d_6) δ 8.04 (d, *J* = 15.5 Hz, 2H), 7.31 (q, *J* = 8.7 Hz, 10H), 6.32 (d, *J* = 15.6 Hz, 2H), 4.23 (d, *J* = 5.7 Hz, 4H), 2.71 (t, *J* = 6.9 Hz, 4H), 1.76 (p, *J* = 3.3 Hz, 8H); ^{13}C NMR (75 MHz, Acetone-d_6) δ 166.24, 147.91, 141.25, 140.27, 131.33 , 130.18, 128.29, 126.83, 118.44, 113.42, 63.86, 34.45, 28.20, 27.71; HRMS (ESI) m/z calcd for $C_{32}H_{32}O_6Cl_2Na^+$ [M + Na] $^+$ 605.1468 found 605.1462.

bis(4-(trifluoromethyl)phenethyl) 3,3'-(4,5-dihydroxy-1,2-phenylene)(2*E*,2'*E*)-diacrylate (35)

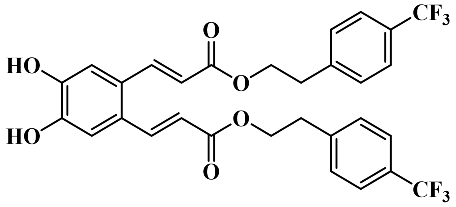

Slight yellow solid (42.1%); mp. 179-183℃; ^{1}H NMR (300 MHz, Acetone-d$_6$) δ 8.02 (d, *J* = 15.7 Hz, 2H), 7.70 – 7.57 (m, 8H), 7.23 (s, 2H), 6.27 (d, *J* = 15.6 Hz, 2H), 4.46 (t, *J* = 6.4 Hz, 4H), 3.14 (t, *J* = 6.4 Hz, 4H); ^{13}C NMR (75 MHz, Acetone-*d*$_6$) δ 166.12, 147.91, 143.43, 140.43, 129.81, 126.79, 125.31 (q, *J* = 5.6 Hz), 125.27, 118.14, 113.33, 64.30, 34.68; HRMS (ESI) m/z calcd for $C_{30}H_{24}O_6F_6Na^+$ [M + Na] $^+$ 617.1359 found 617.1363.

bis(3-(4-(trifluoromethyl)phenyl)propyl)3,3'-(4,5-dihydroxy-1,2-phenylene) (2*E*,2'*E*)-diacrylate (36)

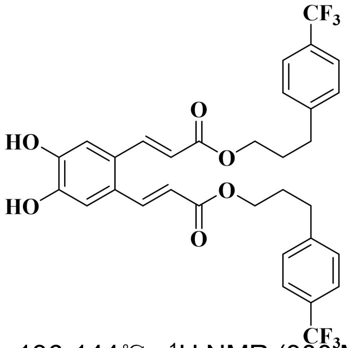

Slight yellow solid (40.6%); mp. 136-144℃; ^{1}H NMR (300 MHz, MeOD) δ 8.06 (d, *J* = 15.8 Hz, 2H), 7.59 – 7.41 (m, 8H), 7.12 (s, 2H), 6.26 (d, *J* = 15.9 Hz, 2H), 4.22 (t, 4H), 2.84 (t, *J* = 6.9 Hz, 4H), 2.11 – 2.01 (m, 4H); ^{13}C NMR (75 MHz, MeOD) δ 166.21, 147.74, 140.50, 140.37, 131.19, 130.22, 128.39, 126.94(q, *J* = 5.6 Hz), 118.45, 113.47, 63.33, 31.27, 30.27; HRMS (ESI) m/z calcd for $C_{32}H_{28}O_6F_6Na^+$ [M + Na] $^+$ 645.1682 found 645.1688.

bis(2,3-dichlorophenethyl)
3,3'-(4,5-dihydroxy-1,2-phenylene)(2*E*,2'*E*)-diacrylate (37a)

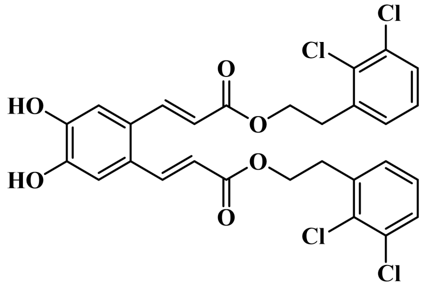

Slight yellow solid (40.0%); mp. 192-198℃; ^{1}H NMR (300 MHz, Acetone-d_6) δ 8.00 (d, J = 15.7 Hz, 2H), 7.56 – 7.30 (m, 6H), 7.24 (s, 2H), 6.26 (d, J = 15.7 Hz, 2H), 4.47 (t, J = 6.6 Hz, 4H), 3.25 (t, J = 6.6 Hz, 4H); ^{13}C NMR (75 MHz, Acetone-d_6) δ 166.02, 147.89, 140.49, 138.63, 132.58, 131.89, 130.06, 129.05, 127.95, 126.80, 118.13, 113.34, 62.74, 33.53; HRMS (ESI) m/z calcd for $C_{28}H_{22}O_6Cl_4Na^+$ [M + Na] $^+$ 617.0063 found 617.0066.

bis(2,4-dichlorophenethyl)
3,3'-(4,5-dihydroxy-1,2-phenylene)(2*E*,2'*E*)-diacrylate (37b)

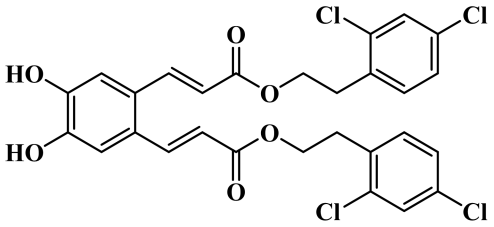

Slight yellow solid (41.7%); mp. 147-150℃; ^{1}H NMR (300 MHz, Acetone-d_6) δ 8.94 (s, 1H), 8.02 (d, J = 15.6 Hz, 2H), 7.51- 7.25 (m, 8H), 6.27 (d, J = 15.2 Hz, 2H), 4.46 (t, J = 6.0 Hz, 4H), 3.19 (t, J = 6.0 Hz, 4H); ^{13}C NMR (75 MHz, Acetone-d_6) δ 166.06, 147.91, 140.48, 135.06, 134.69, 132.72, 128.96, 127.43, 126.82, 118.12, 113.35, 62.90, 32.06; HRMS (ESI) m/z calcd for $C_{28}H_{22}O_6Cl_4Na^+$ [M + Na] $^+$ 617.0063 found 617.0065.

bis(2,5-dichlorophenethyl) 3,3'-(4,5-dihydroxy-1,2-phenylene)(2*E*,2'*E*)-diacrylate (37c)

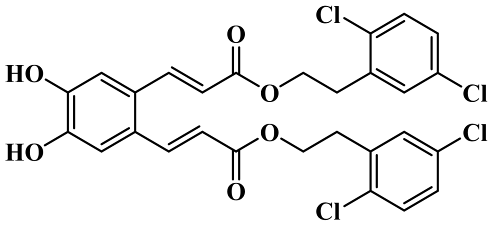

Slight yellow solid (43.2%); mp. 164-170℃; ^{1}H NMR (300 MHz, Acetone-d_6) δ 8.01 (d, J = 15.7 Hz, 2H), 7.51 – 7.38 (m, 4H), 7.28 (dd, J = 8.6, 2.5 Hz, 2H), 7.24 (s, 2H), 6.27 (d, J = 15.7 Hz, 2H), 4.45 (t, J = 6.6 Hz, 4H), 3.19 (t, J = 6.6 Hz, 4H); ^{13}C NMR (75 MHz, Acetone-d_6) δ 166.02, 147.91, 140.62, 138.13, 132.51, 132.36, 131.14, 130.88, 128.33, 126.84, 118.14, 113.40, 62.84, 32.48; HRMS (ESI) m/z calcd for $C_{28}H_{22}O_6Cl_4Na^+$ $[M + Na]^+$ 617.0063 found 617.0069.

bis(2,6-dichlorophenethyl) 3,3'-(4,5-dihydroxy-1,2-phenylene)(2*E*,2'*E*)-diacrylate (**37d**)

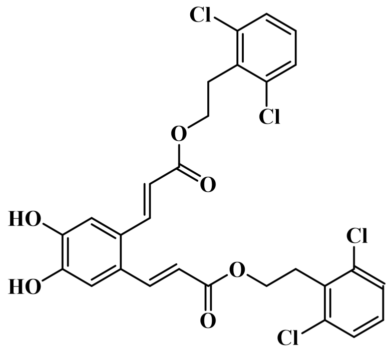

Slight yellow solid (39.6%); mp. 132-136℃; ^{1}H NMR (300 MHz, Acetone-d_6) δ 8.96 (s, 1H), 8.04 (d, J = 15.7 Hz, 2H), 7.46 (d, J = 8.1 Hz, 4H), 7.38 – 7.23 (m, 4H), 6.26 (d, J = 15.7 Hz, 2H), 4.46 (t, J = 6.7 Hz, 4H), 3.39 (t, J = 6.5 Hz, 4H); ^{13}C NMR (75 MHz, Acetone-d_6) δ 166.02, 147.89, 140.54, 135.69, 133.95, 129.14, 128.58, 126.92, 118.27, 113.38, 61.80, 30.47; HRMS (ESI) m/z calcd for $C_{28}H_{22}O_6Cl_4Na^+$ $[M + Na]^+$ 617.0063 found 617.0061.

bis(3,4-dichlorophenethyl) 3,3'-(4,5-dihydroxy-1,2-phenylene)(2*E*,2'*E*)-diacrylate (37e)

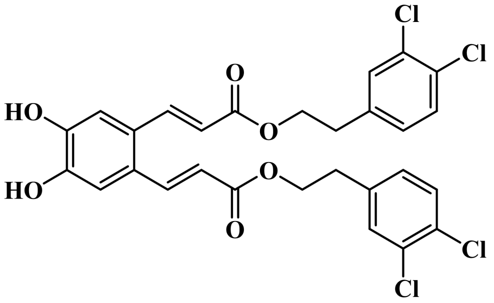

Slight yellow solid (37.2%); mp. 192-198℃; ^{1}H NMR (300 MHz, Acetone-d_6) δ 8.03 (dd, *J* = 15.7, 2.7 Hz, 2H), 7.59 – 7.54 (m, 4H), 7.37 (dd, *J* = 8.3, 2.1 Hz, 2H), 7.26 (d, *J* = 2.6 Hz, 2H), 6.30 (d, *J* = 15.6 Hz, 2H), 4.45 (t, *J* = 6.6 Hz, 4H), 3.08 (t, *J* = 6.5 Hz, 4H); ^{13}C NMR (75 MHz, Acetone-d_6) δ 166.04, 147.90, 140.52, 139.73, 131.64, 131.08, 130.51, 129.82, 129.34, 126.84, 118.17, 113.46, 64.19, 33.96; HRMS (ESI) m/z calcd for $C_{28}H_{22}O_6Cl_4Na^+$ [M + Na] $^+$ 617.0063 found 617.0067.

bis(3,5-dichlorophenethyl) 3,3'-(4,5-dihydroxy-1,2-phenylene)(2*E*,2'*E*)-diacrylate (37f)

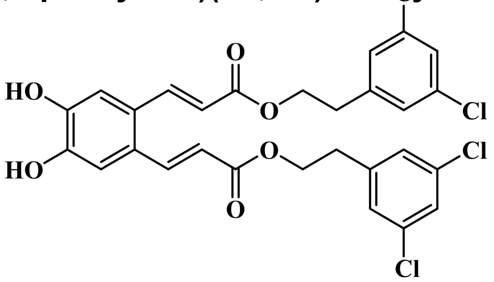

Slight yellow solid (36.5%); mp. 202-206℃; ^{1}H NMR (300 MHz, Acetone-d_6) δ 8.03 (d, *J* = 15.6 Hz, 2H), 7.40 - 7.37 (m, 6H), 7.26 (s, 2H), 6.30 (d, *J* = 15.7 Hz, 2H), 4.46 (t, *J* = 6.6 Hz, 4H), 3.09 (t, *J* = 6.6 Hz, 4H); ^{13}C NMR (75 MHz, Acetone-d_6) δ 165.99, 147.88, 142.82, 140.62, 134.51, 127.85, 126.87, 126.36, 118.17, 113.52, 63.98, 34.26; HRMS (ESI) m/z calcd for $C_{28}H_{22}O_6Cl_4Na^+$ [M + Na] $^+$ 617.0063 found 617.0064.

bis((*R*)-1-phenylpropan-2-yl) 3,3'-(4,5-dihydroxy-1,2-phenylene)(2*E*,2'*E*)-diacrylate (38a)

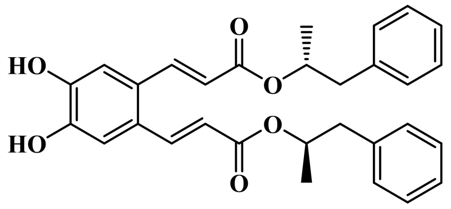

Slight yellow solid (45.2%); mp. 120-124℃; ^{1}H NMR (300 MHz, Acetone-d_6) δ 8.76 (s, 1H), 7.99 (d, *J* = 15.7 Hz, 1H), 7.32 – 7.26 (m, 4H), 7.22 (s, 1H), 6.24 (d, *J* = 15.7 Hz, 1H), 5.26 – 5.15 (m, 1H), 3.03 – 2.86 (m, 2H), 1.28 (d, *J* = 6.2 Hz, 3H); ^{13}C NMR (75 MHz, Acetone-d_6) δ 166.50, 148.66, 140.91, 138.90, 130.34, 129.14, 127.73, 127.21, 119.67, 114.26, 72.34, 42.78, 19.90; HRMS (ESI) m/z calcd for $C_{30}H_{30}O_6Na^+$ [M + Na] $^+$ 509.1935 found 509.1936.

bis((*S*)-1-phenylpropan-2-yl) 3,3'-(4,5-dihydroxy-1,2-phenylene)(2*E*,2'*E*)-diacrylate (**38b**)

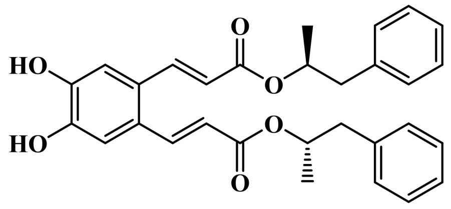

Slight yellow solid (42.8%); mp. 120-124℃; ^{1}H NMR (300 MHz, Acetone-d_6) δ 8.77 (s, 2H), 8.01 (d, *J* = 15.7 Hz, 2H), 7.37 – 7.26 (m, 10H), 6.26 (d, *J* = 15.7 Hz, 2H), 5.22 (h, 2H), 2.95 (h, 4H), 1.30 (d, *J* = 6.2 Hz, 6H); ^{13}C NMR (75 MHz, Acetone-d_6) δ 165.63, 147.80, 140.04, 138.04, 129.47, 128.28, 126.87, 126.35, 118.81, 113.40, 71.47, 41.92, 19.04; HRMS (ESI) m/z calcd for $C_{30}H_{30}O_6Na^+$ [M + Na] $^+$ 509.1935 found 509.1934.

bis(1-phenylpropan-2-yl) 3,3'-(4,5-dihydroxy-1,2-phenylene)(2*E*,2'*E*)-diacrylate (38c)

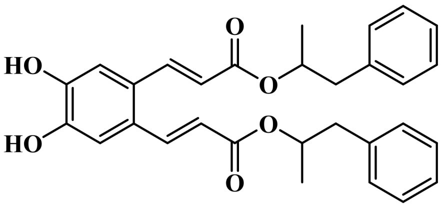

Slight yellow solid (48.3%); ^{1}H NMR (300 MHz, Acetone-d_6) δ 8.76 (s, 2H), 8.00 (d, *J* = 15.7 Hz, 2H), 7.33 – 7.26 (m, 8H), 7.23 (s, 2H), 6.25 (d, *J* = 15.7 Hz, 2H), 5.26 – 5.16(m, 2H), 3.04 – 2.87 (m, 4H), 1.29 (d, *J* = 6.2 Hz, 6H); ^{13}C NMR (75 MHz, Acetone-d_6) δ 166.52, 148.68, 140.93, 138.93, 130.36, 129.17, 127.75, 127.23, 119.70, 114.29, 72.36, 42.81, 19.93; HRMS (ESI) m/z calcd for $C_{30}H_{30}O_6Na^+$ [M + Na] $^+$ 509.1935 found 509.1933.

bis((*R*)-1-(p-tolyl)propan-2-yl)3,3'-(4,5-dihydroxy-1,2-phenylene)(2*E*,2'*E*)-diacrylate (39a)

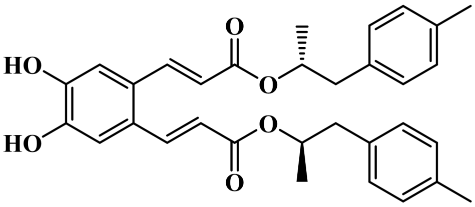

Slight yellow solid (43.9%); mp. 143-148℃；^{1}H NMR (300 MHz, Acetone-d_6) δ 8.00 (d, *J* = 15.7 Hz, 2H), 7.22 – 7.09 (m, 10H), 6.23 (d, *J* = 15.6 Hz, 2H), 5.17 (h, *J* = 5.7, 5.1 Hz, 2H), 2.98-2.79 (m, 4H), 2.26 (s, 6H), 1.26 (d, *J* = 5.7 Hz, 6H); ^{13}C NMR (75 MHz, Acetone-d_6) δ 166.69, 148.07, 140.47, 135.71, 134.55, 129.03, 128.63, 126.54, 117.92, 112.82, 72.00, 41.41, 19.71, 18.49; HRMS (ESI) m/z calcd for $C_{32}H_{34}O_6Na^+$ [M + Na] $^+$ 537.2253 found 537.2259.

bis((*S*)-1-(p-tolyl)propan-2-yl)3,3'-(4,5-dihydroxy-1,2-phenylene)(2*E*,2'*E*)-diacrylate (39b)

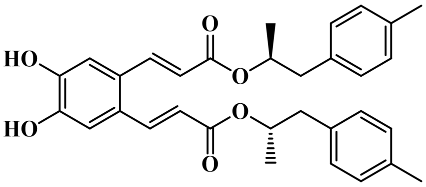

Slight yellow solid (41.1%); mp. 173-148℃; ^{1}H NMR (300 MHz, MeOD) δ 7.98 (d, *J* = 15.7, 2H), 7.12 (m, 10H), 6.20 (d, *J* = 15.6, 2H), 5.18 (h, *J* = 5.7, 5.1, 2H), 2.99-2.79 (m , 4H) , 2.29 (s, 6H) 1.28 (d, *J* = 5.7 Hz, 6H); ^{13}C NMR (75 MHz, MeOD) δ 166.69, 148.06, 140.47, 135.70, 134.54, 129.03, 128.63, 126.54, 117.92, 112.83, 72.00, 41.41, 19.73, 18.49; HRMS (ESI) m/z calcd for $C_{32}H_{34}O_6Na^+$ [M + Na] $^+$ 537.2253 found 537.2257.

bis(1-(p-tolyl)propan-2-yl) 3,3'-(4,5-dihydroxy-1,2-phenylene)(2*E*,2'*E*)-diacrylate (39c)

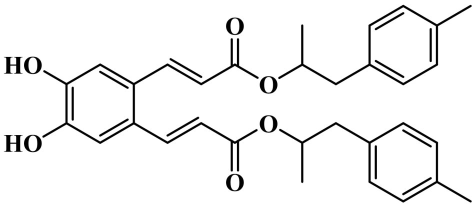

Slight yellow oil (48.6%); ^{1}H NMR (300 MHz, MeOD) δ 8.01 (d, *J* = 15.7 Hz, 2H), 7.16 – 7.09 (m, 10H), 6.26 (d, *J* = 15.7 Hz, 2H), 5.22 - 5.15 (h, 2H), 2.99 - 2.79 (m, 4H), 1.30 (d, *J* = 6.2 Hz, 6H); ^{13}C NMR (75 MHz, MeOD) δ 165.63, 147.80, 140.04, 138.04, 129.47, 128.28, 126.87, 126.35, 118.81, 113.40, 71.47, 41.92, 20.01, 19.04; HRMS (ESI) m/z calcd for $C_{32}H_{34}O_6Na^+$ [M + Na] $^+$ 537.2253 found 537.2257.

bis((*R*)-1-(4-chlorophenyl)propan-2-yl)3,3'-(4,5-dihydroxy-1,2-phenylene)(2*E*,2'*E*)-diacrylate (**40a**)

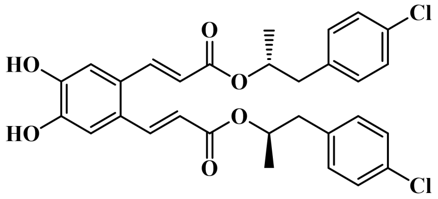

Slight yellow solid (44.1%); mp. 173-176℃; ^{1}H NMR (300 MHz, MeOD) δ 7.99 (d, *J* = 15.6 Hz, 2H), 7.31 – 7.24 (m, 8H), 7.11 (s, 2H), 6.22 (d, *J* = 15.6 Hz, 2H), 5.22 (m, 2H), 2.94 (m, 4H), 1.31 (d, *J* = 6.3 Hz, 6H); ^{13}C NMR (75 MHz, Acetone-d_6) δ 165.65, 147.81, 140.15, 137.01, 131.74, 131.25, 128.29, 126.76, 118.59, 113.30, 71.19, 41.07, 19.02; HRMS (ESI) m/z calcd for $C_{30}H_{28}O_6Cl_2Na^+$ [M + Na] $^+$ 577.1155 found 577.1151.

bis((*S*)-1-(4-chlorophenyl)propan-2-yl)3,3'-(4,5-dihydroxy-1,2-phenylene)(2*E*,2'*E*)-diacrylate (**40b**)

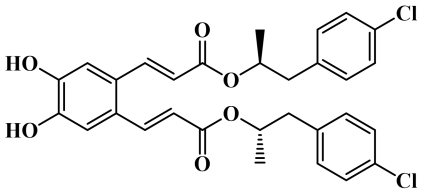

Slight yellow solid (44.6%); mp. 173-176℃; ^{1}H NMR (300 MHz, MeOD) δ 7.99 (d, *J* = 15.6 Hz, 2H), 7.31 – 7.24 (m, 8H), 7.11 (s, 2H), 6.21 (d, *J* = 15.6 Hz, 2H), 5.22 (m, 2H), 3.01 – 2.86 (m, 4H), 1.31 (d, *J* = 6.3 Hz, 6H); ^{13}C NMR (75 MHz, MeOD) δ 166.64, 148.12, 140.66, 136.54, 132.05, 130.81, 128.11, 126.58, 117.85, 112.94, 71.59, 41.06, 18.56; HRMS (ESI) m/z calcd for $C_{30}H_{28}O_6Cl_2Na^+$ [M + Na] $^+$ 577.1155 found 577.1152.

bis(1-(4-chlorophenyl)propan-2-yl)
3,3'-(4,5-dihydroxy-1,2-phenylene)(2*E*,2'*E*)-diacrylate (40c)

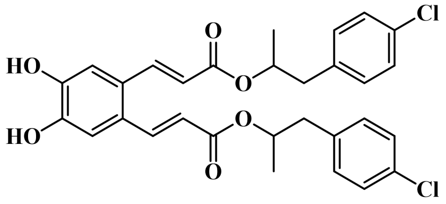

Slight yellow oil (40.3%); ^{1}H NMR (300 MHz, Acetone-d_6) δ 8.87 (s, 2H), 8.04-7.94 (m, 2H), 7.34 – 7.20 (m, 10H), 6.32-6.19 (m, 2H), 5.18 (h, 2H), 2.95 (h, 4H), 1.29 (d, *J* = 6.2 Hz, 6H); ^{13}C NMR (75 MHz, Acetone-d_6) δ 165.63, 147.80, 140.04, 138.04, 129.47, 128.28, 126.87, 126.35, 118.81, 113.40, 71.47, 41.92, 19.04; HRMS (ESI) m/z calcd for $C_{30}H_{28}O_6Cl_2Na^+$ [M + Na] $^+$ 577.1155 found 577.1156.

Figure S1 Supplementary Data on the Validation of WX006's Tumor Growth Inhibitory Efficacy

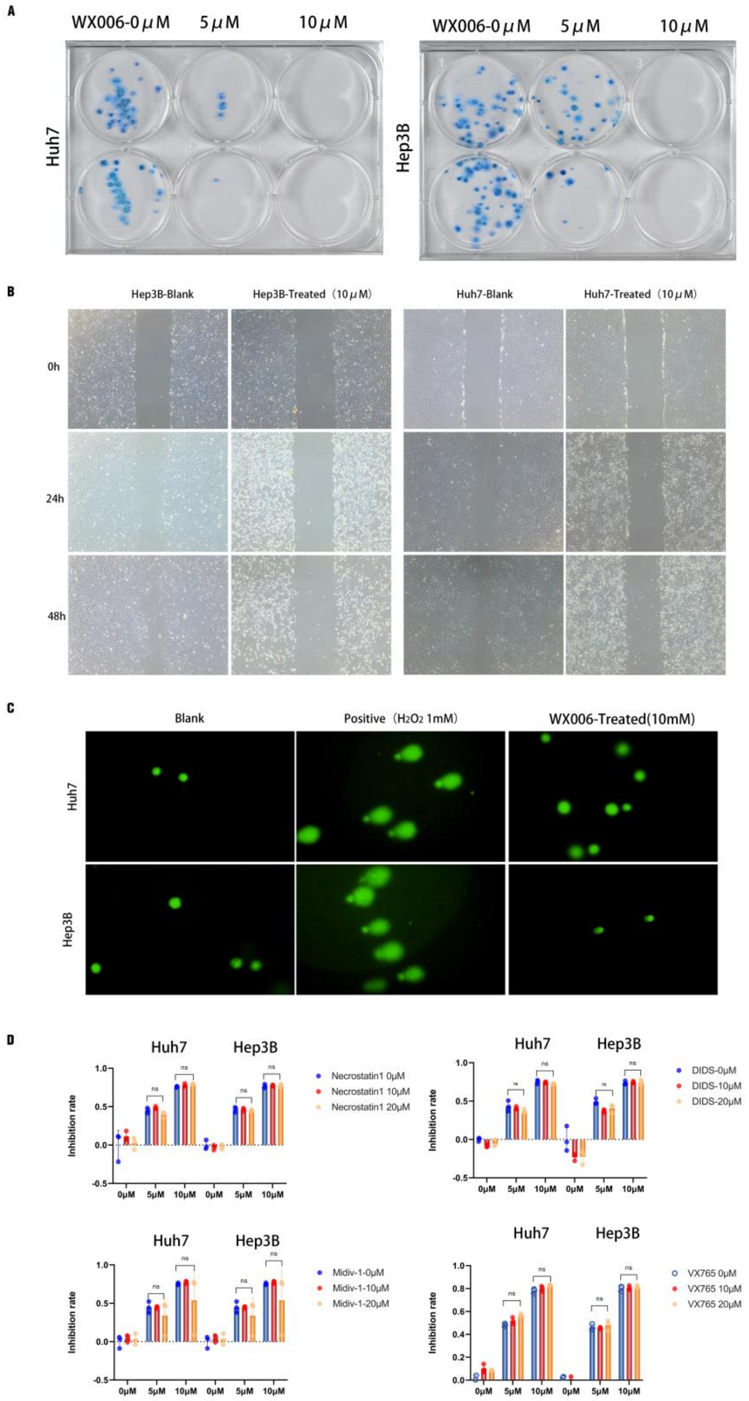

Figure S2 Supplementary Data on the Transcriptomic Effects of WX006

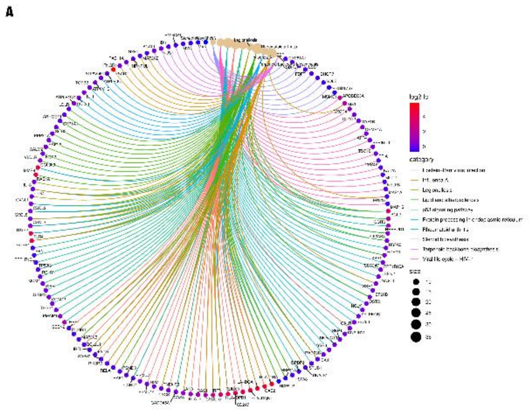

Figure S3 Supplementary Data on the Endoplasmic Reticulum Function Studies of WX006

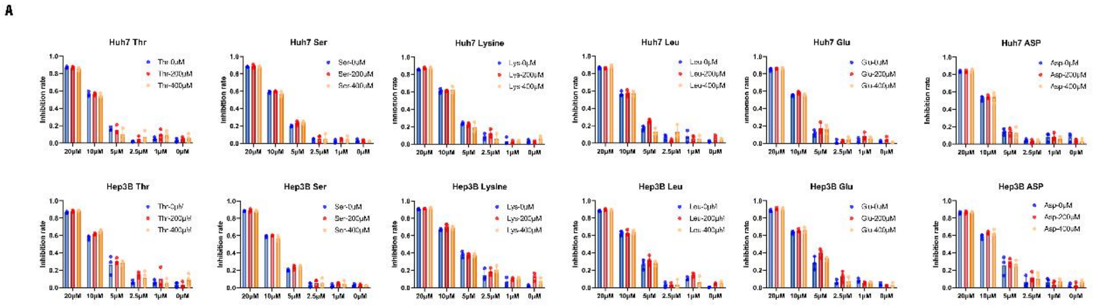

Figure S4 Supplementary Data on WX006-Induced Disruption of Tumor Cell Metabolism

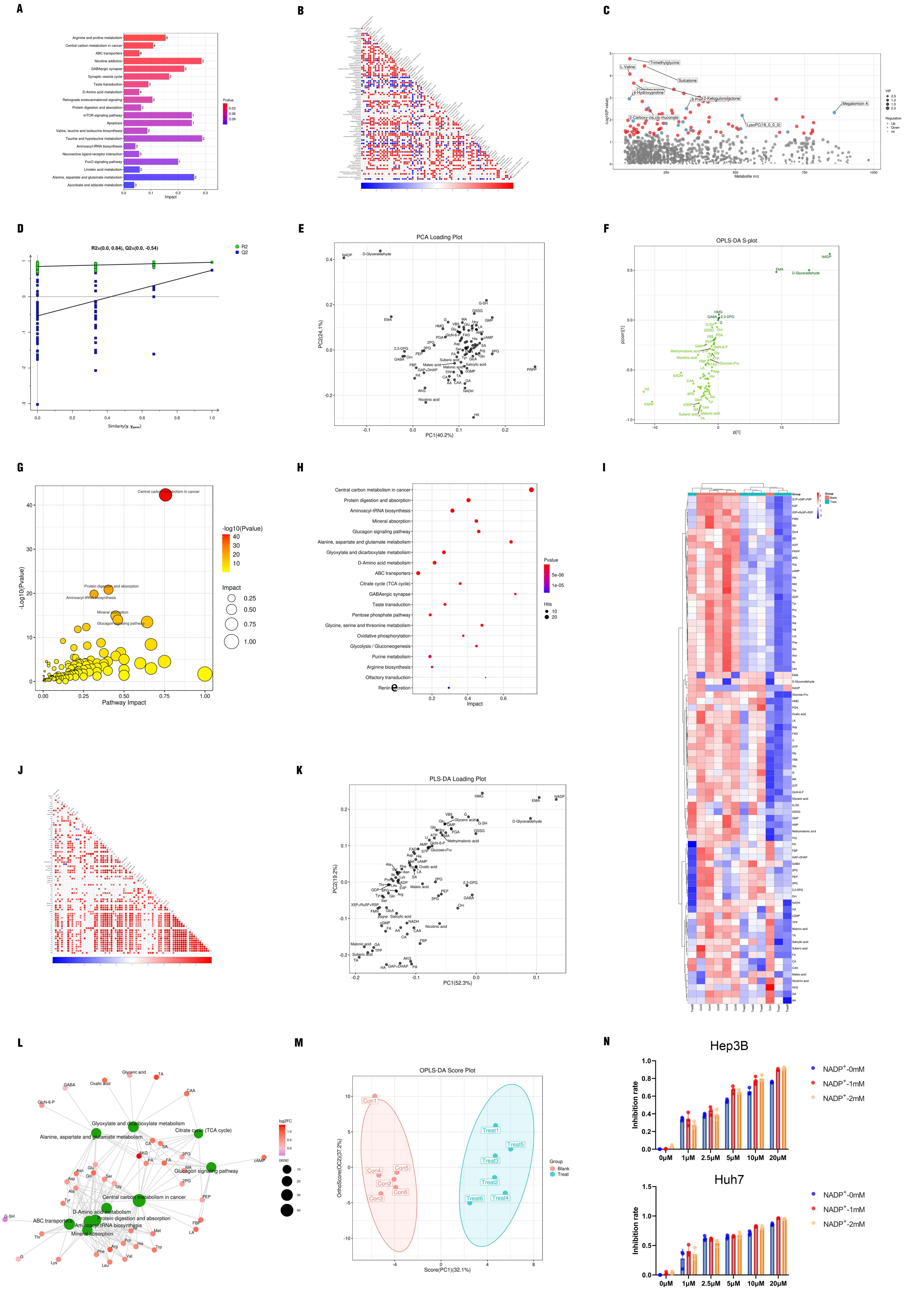

Figure S5 Supplementary Data on the ferroptosis and cuproptosis Studies of WX006

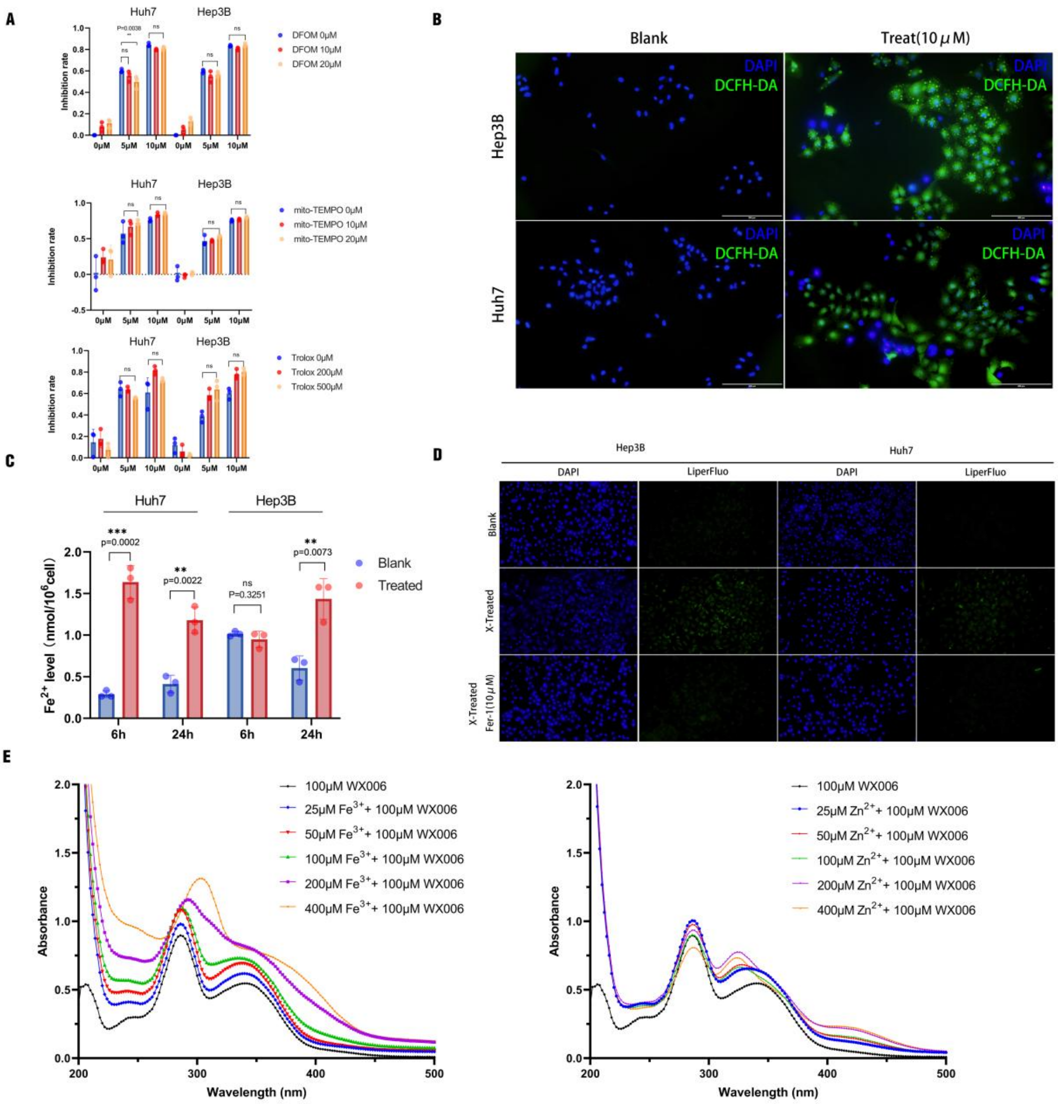

Figure S6 Supplementary Data on the Inhibitory Effects of WX006 on the NDUFS2 Subunit

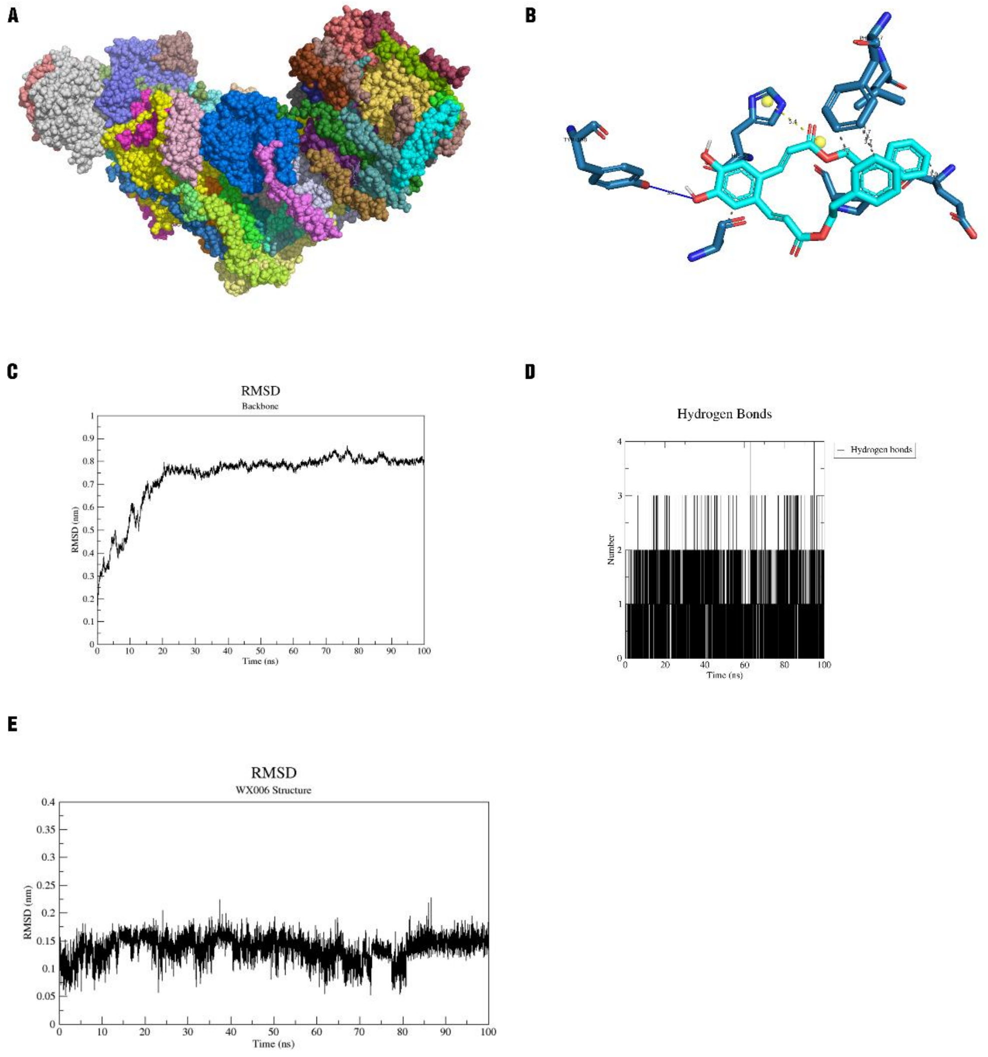

Figure S7 Supplementary Data on the In Vivo Tumor Growth Inhibition by WX006

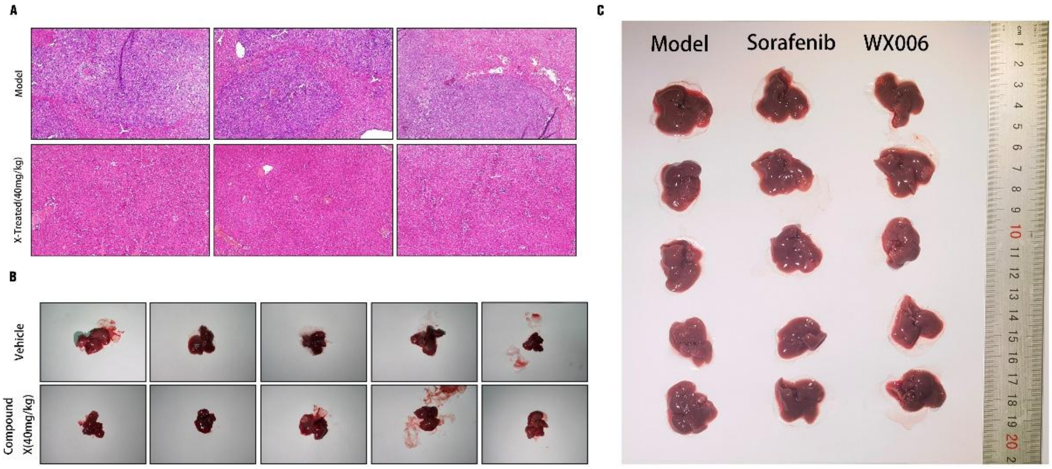

Figure S8 Supplementary Data on the ^{1}H-NMR and ^{13}C-NMR Spectra of WX006 and other derivatives

^{1}H NMR Spectrum of **WX006** (300 MHz, MeOD)

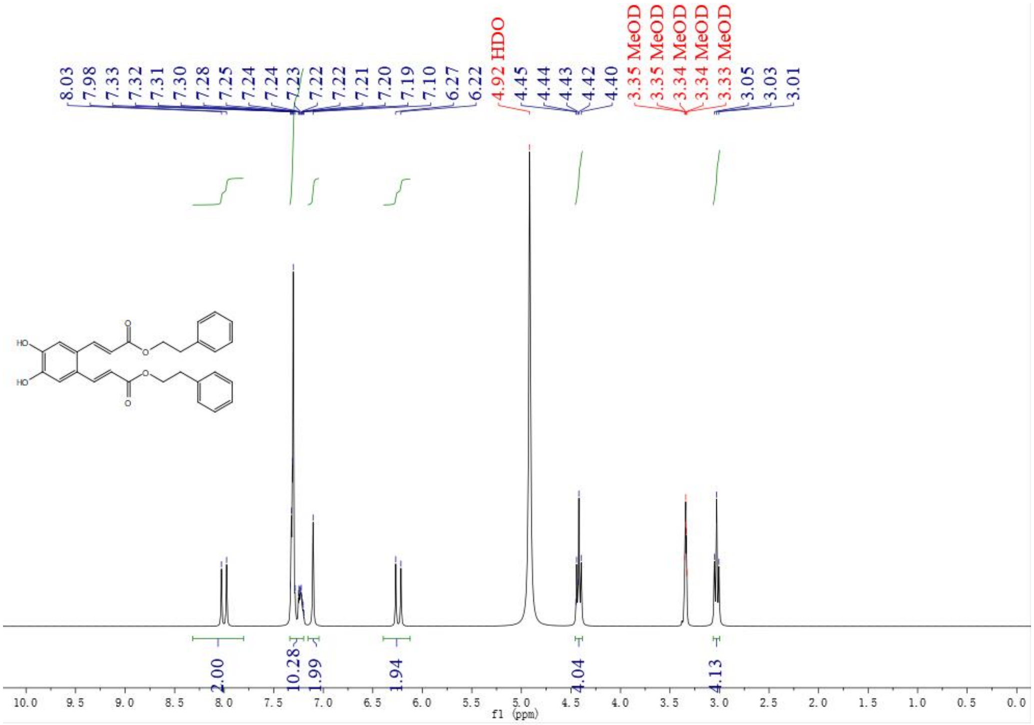

^{13}C NMR Spectrum of **WX006** (75 MHz, MeOD)

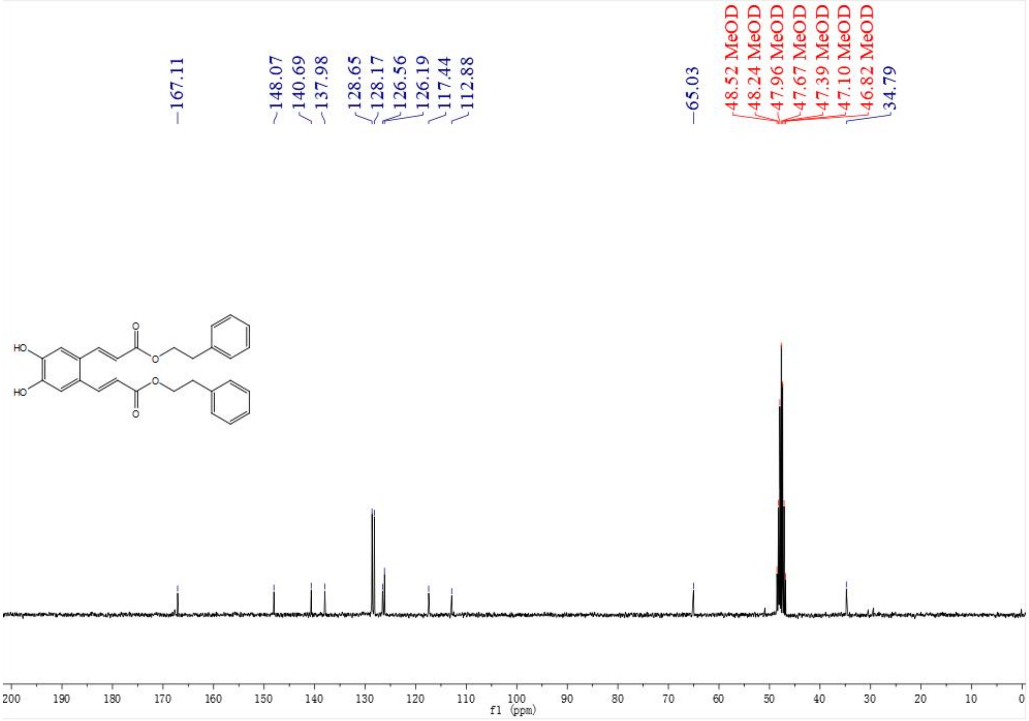

^{1}H NMR Spectrum of **27** (300 MHz, Acetone-d_6)

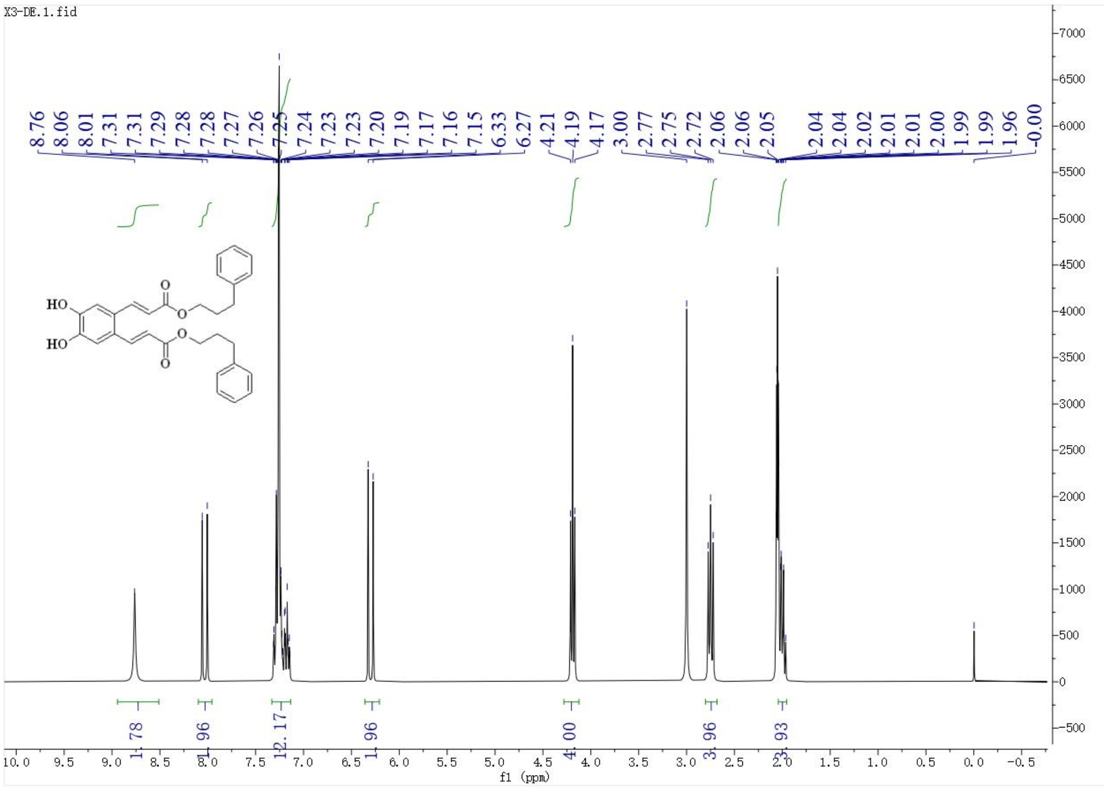

^{13}C NMR Spectrum of **27** (75 MHz, Acetone-d_6)

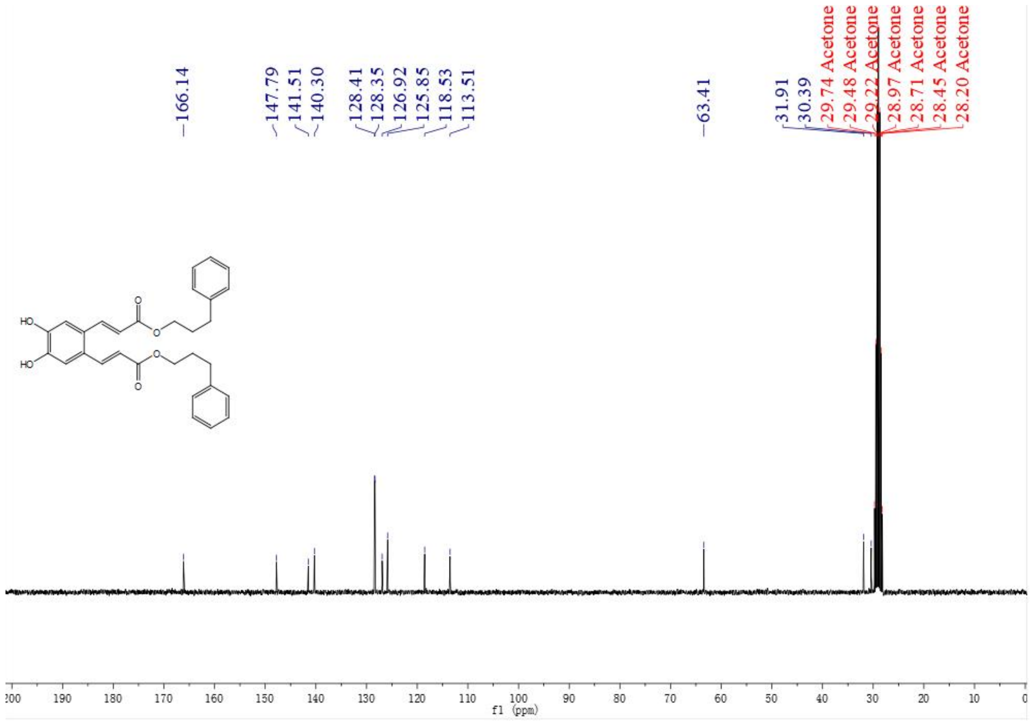

^{1}H NMR Spectrum of **28** (300 MHz, Acetone-d_6)

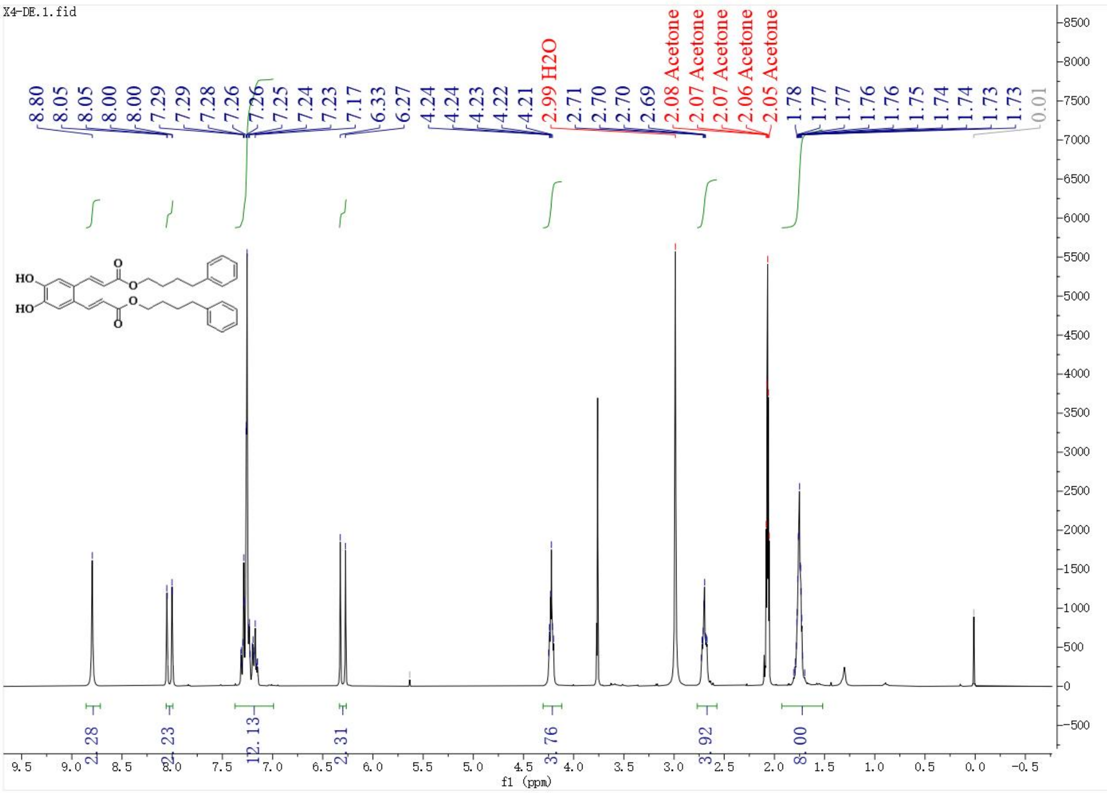

^{13}C NMR Spectrum of **28** (75 MHz, Acetone-d_6)

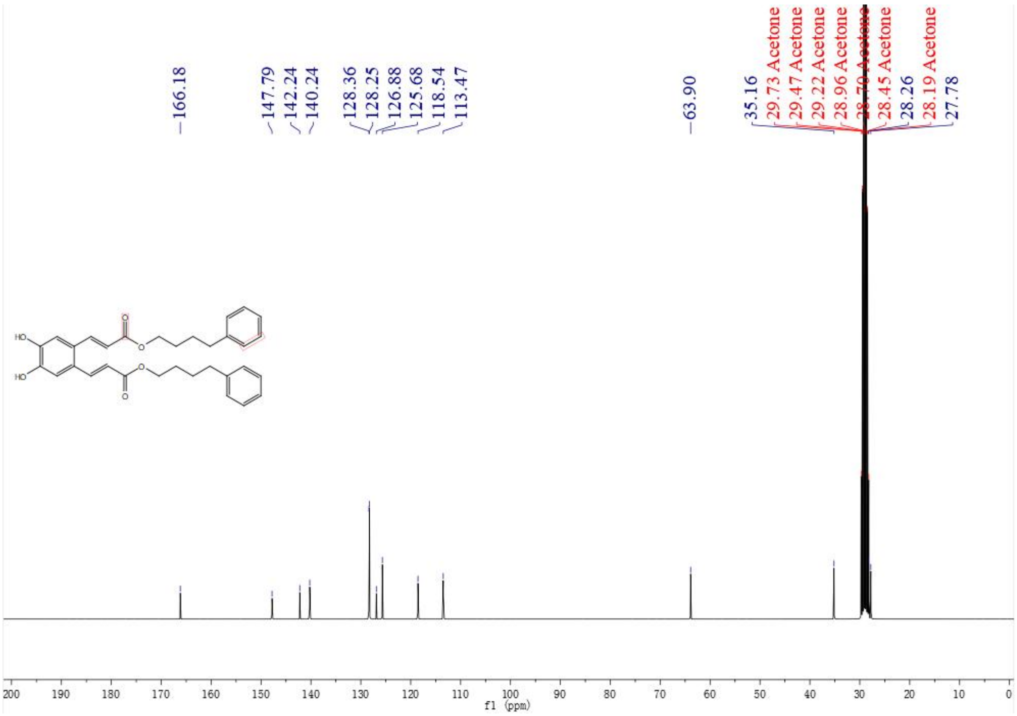

^{1}H NMR Spectrum of **29** (300 MHz, Acetone-d_6)

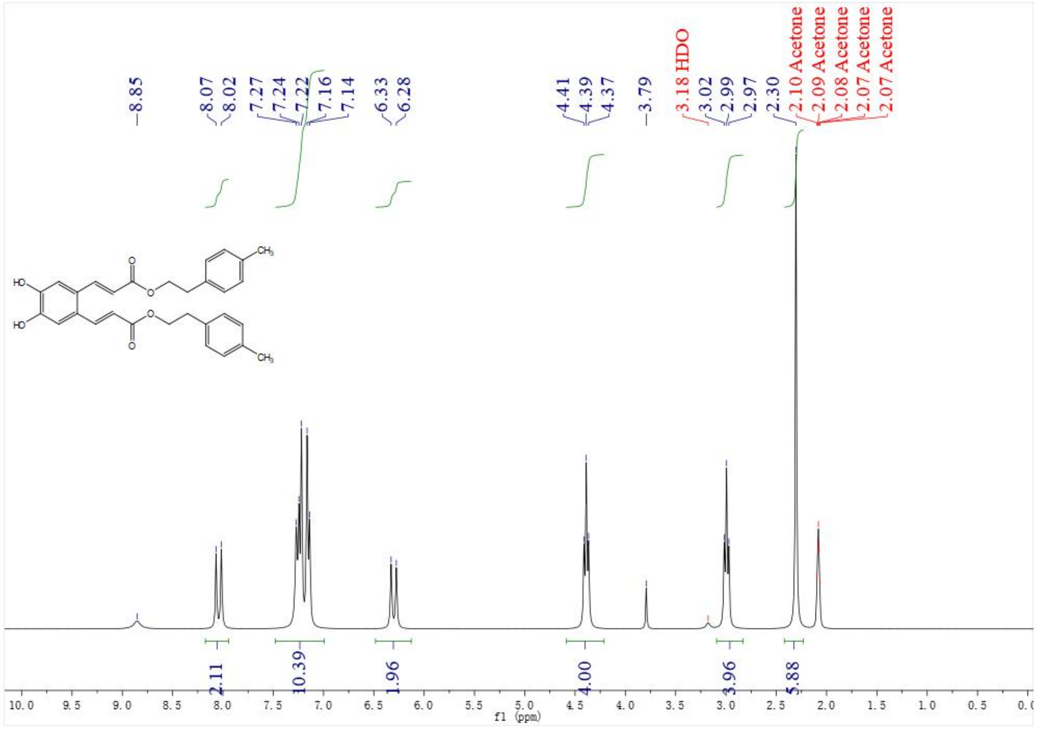

^{13}C NMR Spectrum of **29** (75 MHz, Acetone-d_6)

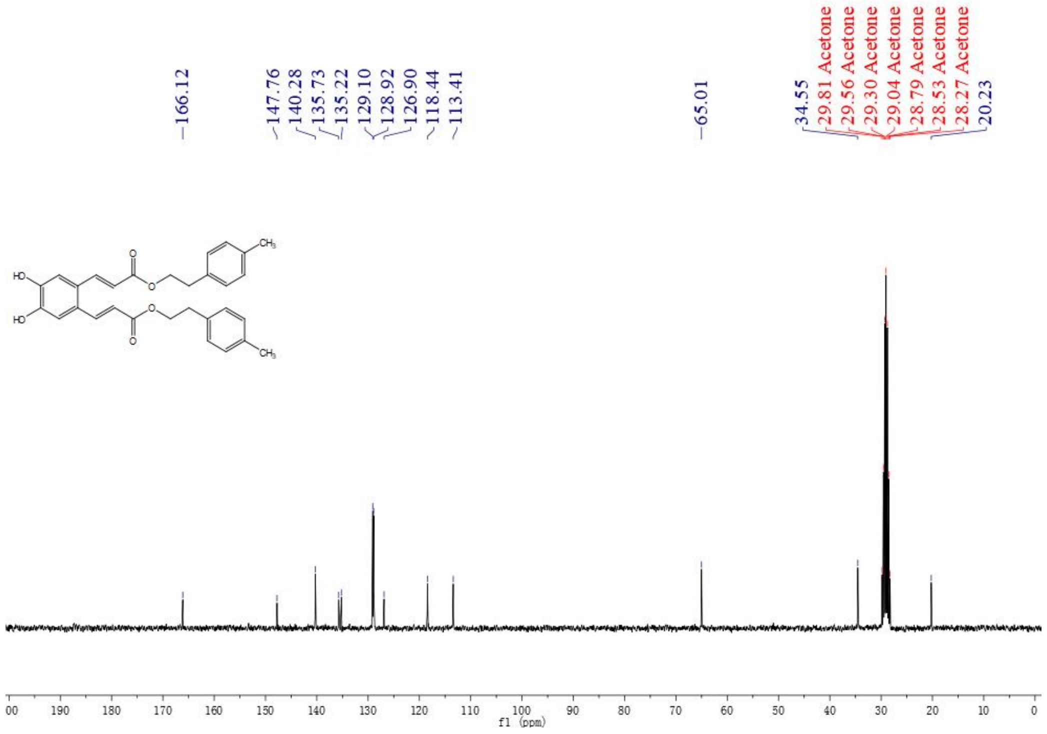

^{1}H NMR Spectrum of **30** (300 MHz, Acetone-d_6)

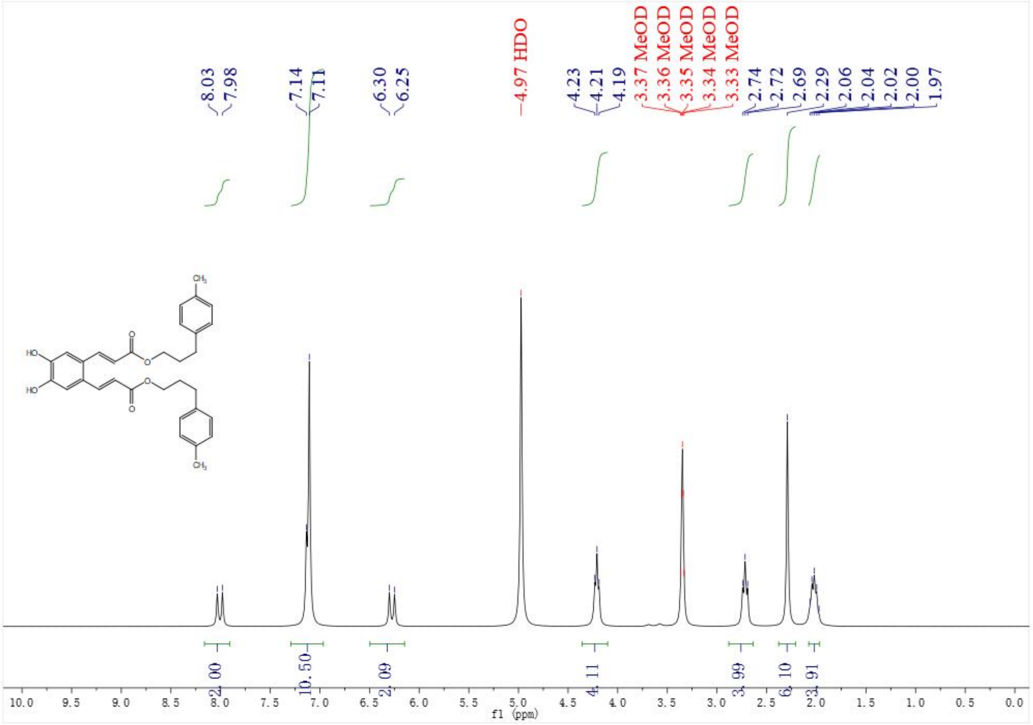

^{13}C NMR Spectrum of **30** (75 MHz, Acetone-d_6)

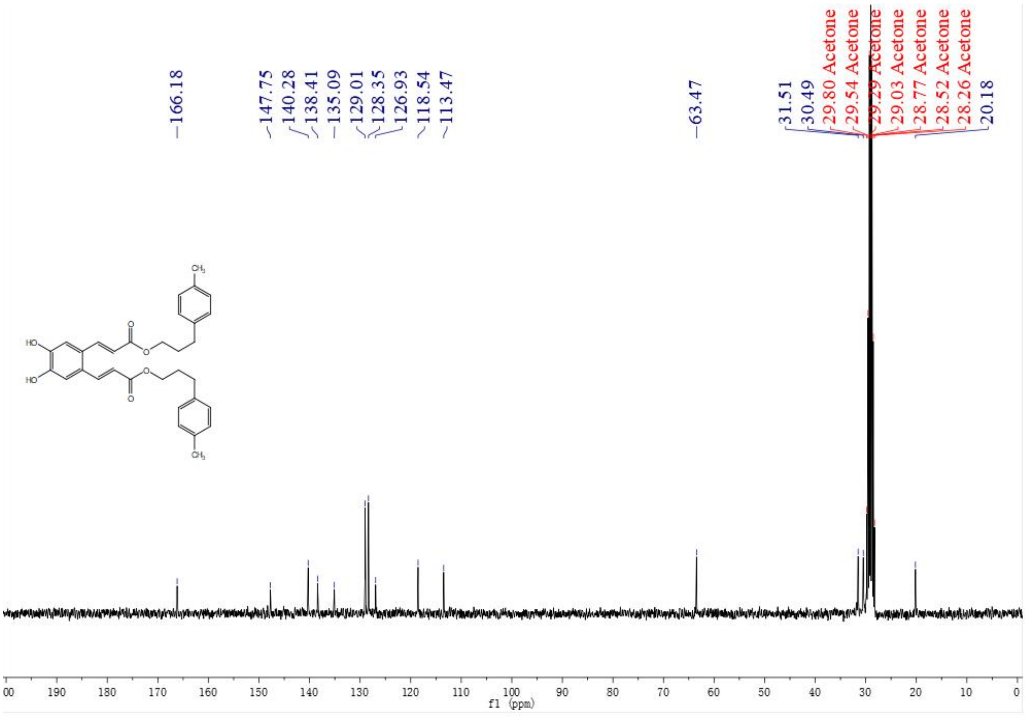

^{1}H NMR Spectrum of **31** (300 MHz, Acetone-d_6)

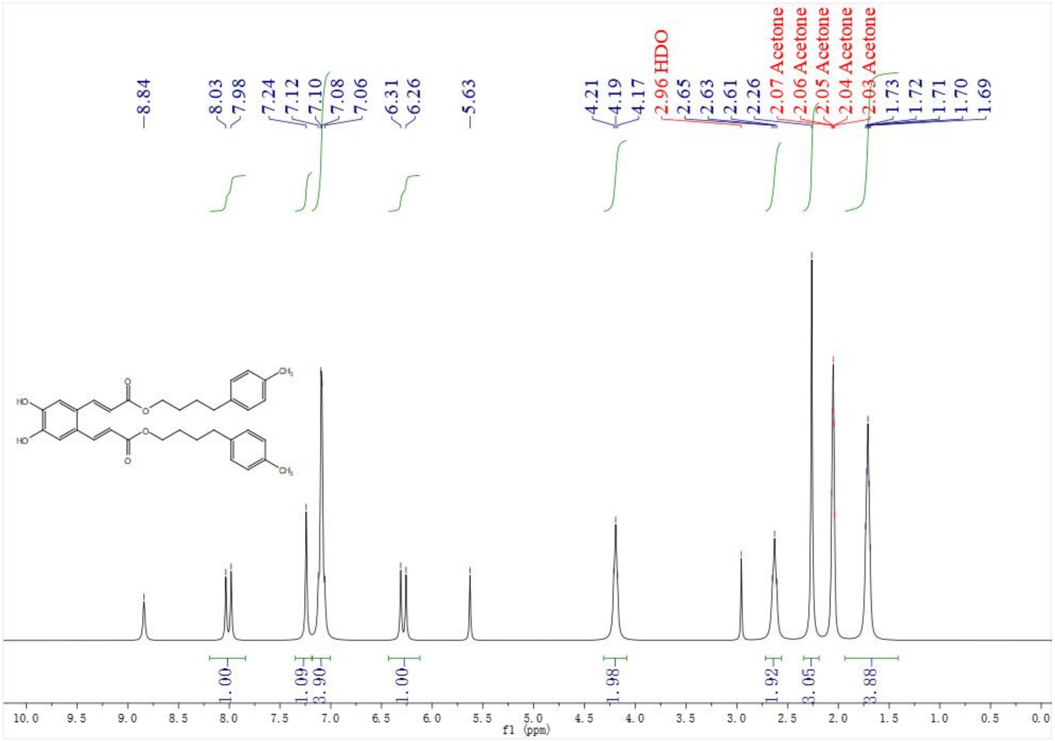

^{13}C NMR Spectrum of **31** (75 MHz, Acetone-d_6)

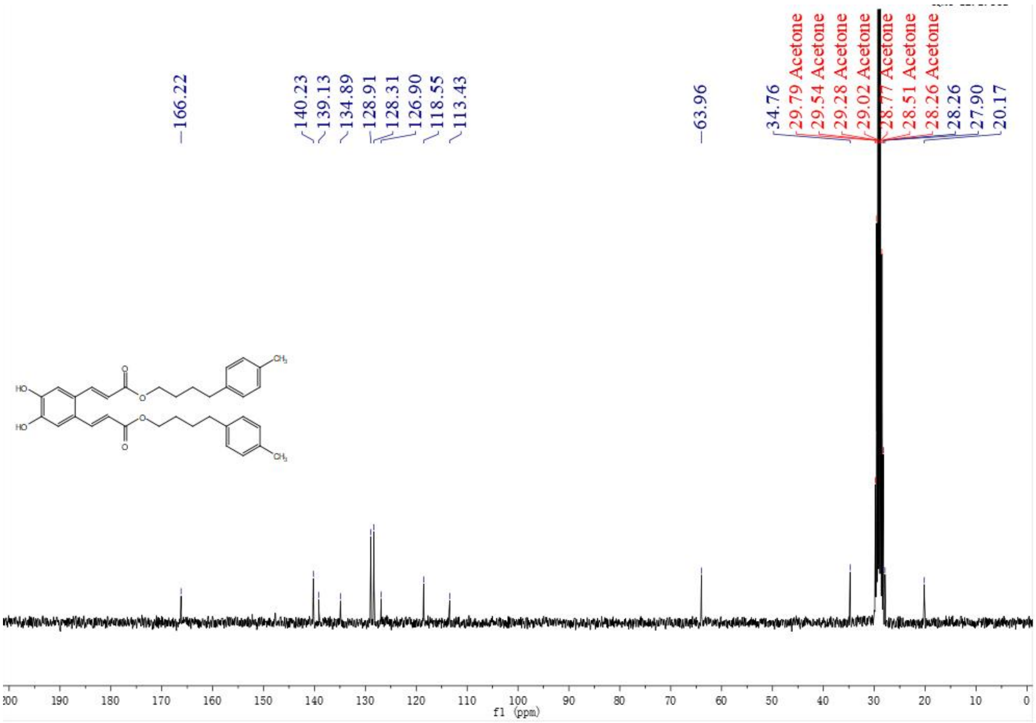

^{1}H NMR Spectrum of **32a** (300 MHz, Acetone-d_6)

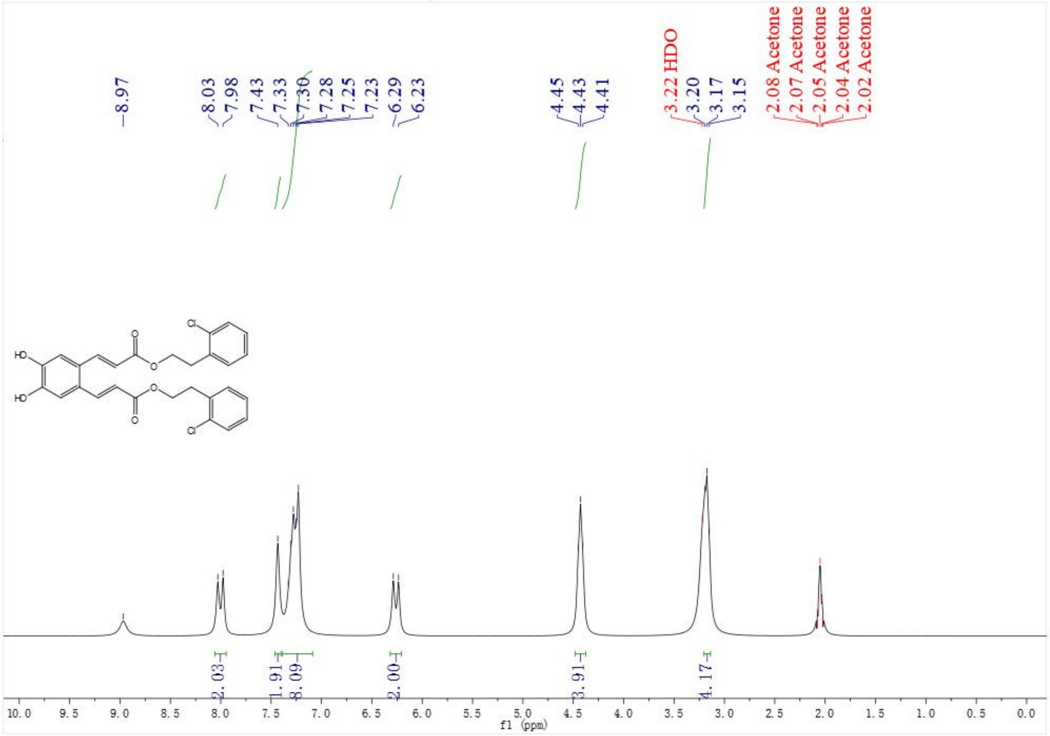

^{13}C NMR Spectrum of **32a** (75 MHz, Acetone-d_6)

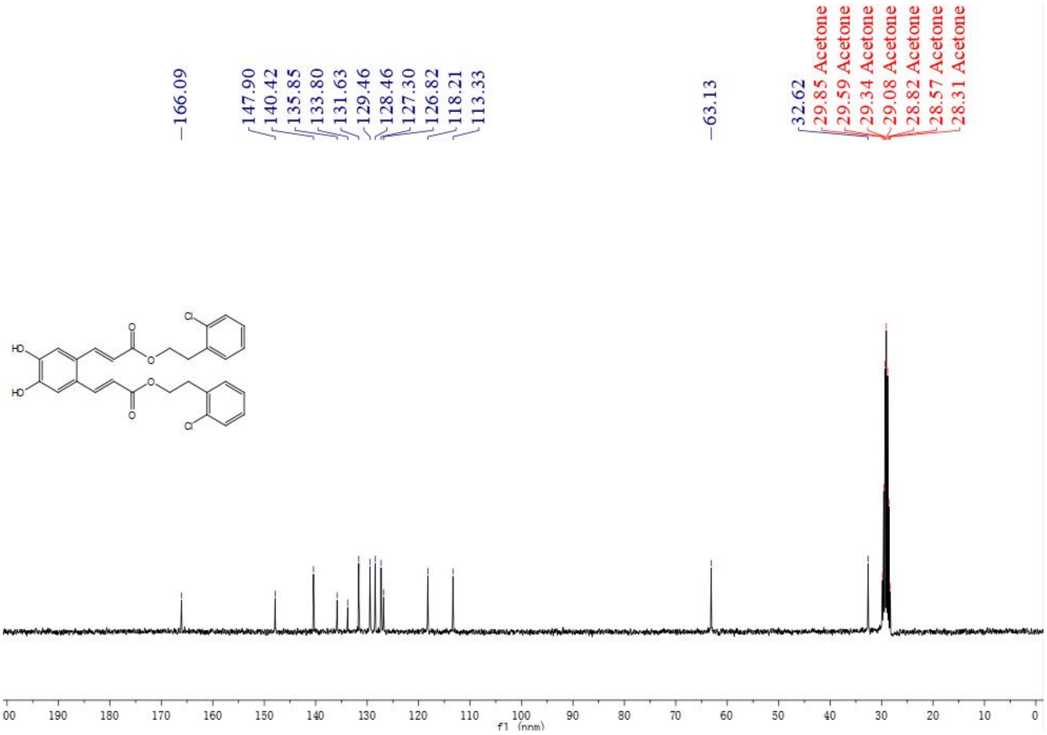

^{1}H NMR Spectrum of **32b** (300 MHz, Acetone-d_6)

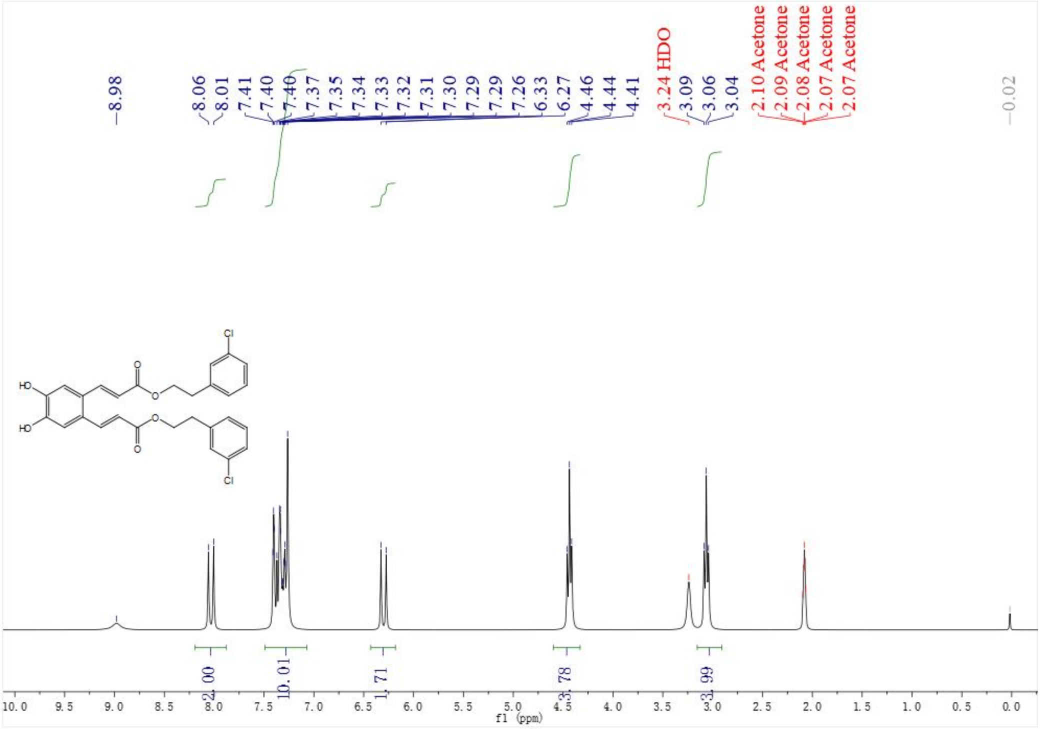

^{13}C NMR Spectrum of **32b** (75 MHz, Acetone-d_6)

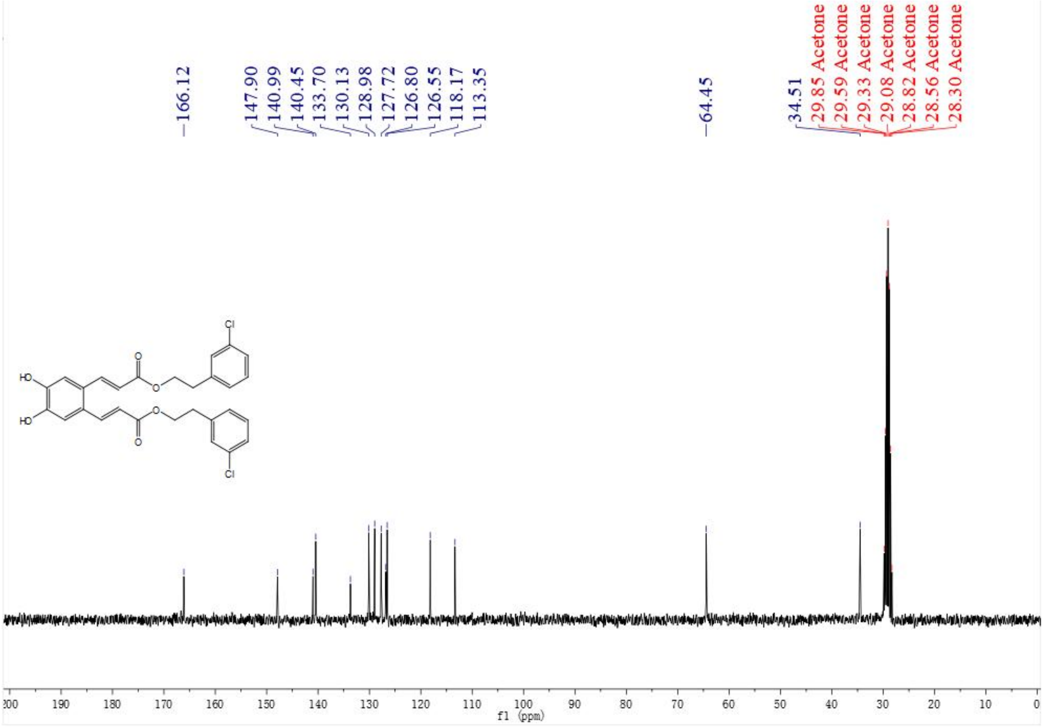

^{1}H NMR Spectrum of **32c** (300 MHz, Acetone-d_6)

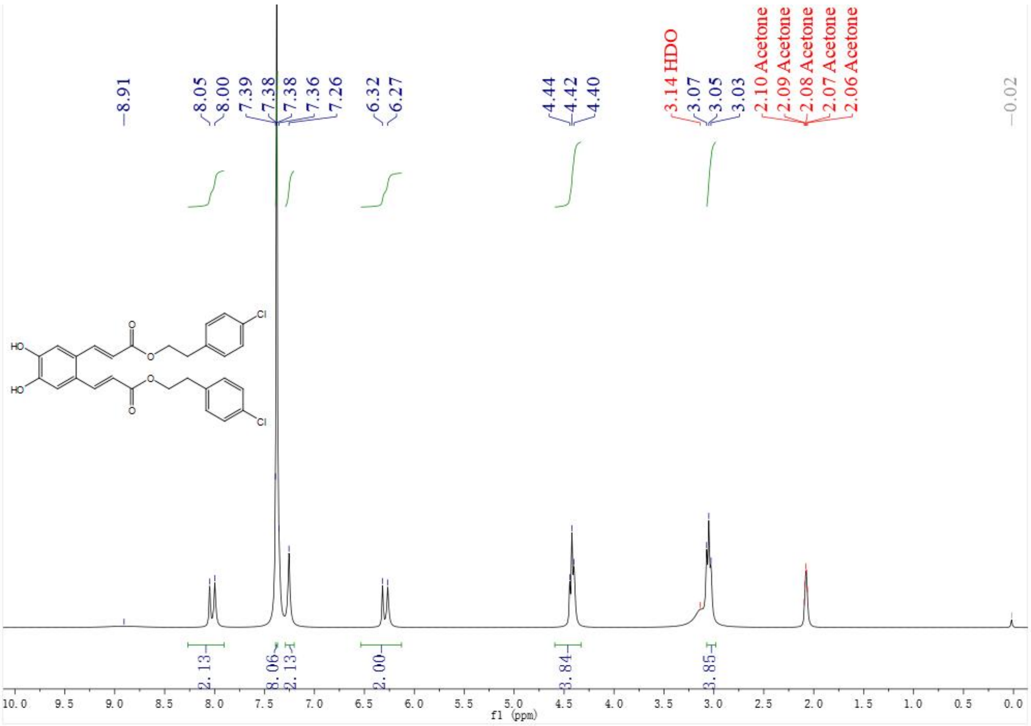

^{13}C NMR Spectrum of **32c** (75 MHz, Acetone-d_6)

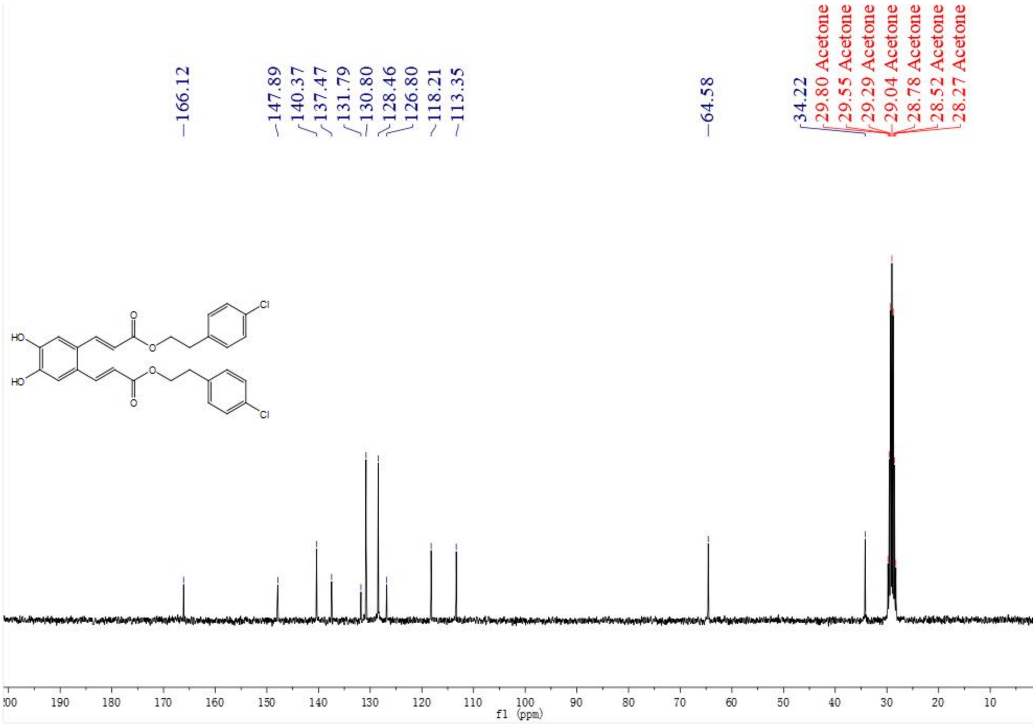

^{1}H NMR Spectrum of **33** (300 MHz, Acetone-d_6)

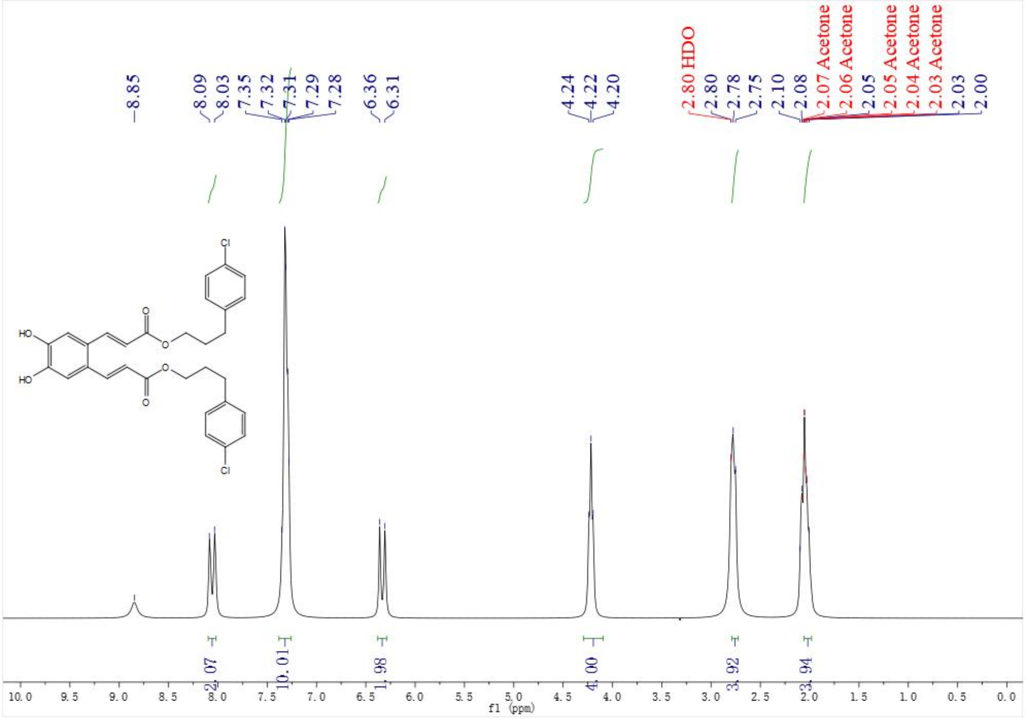

^{13}C NMR Spectrum of **33** (75 MHz, Acetone-d_6)

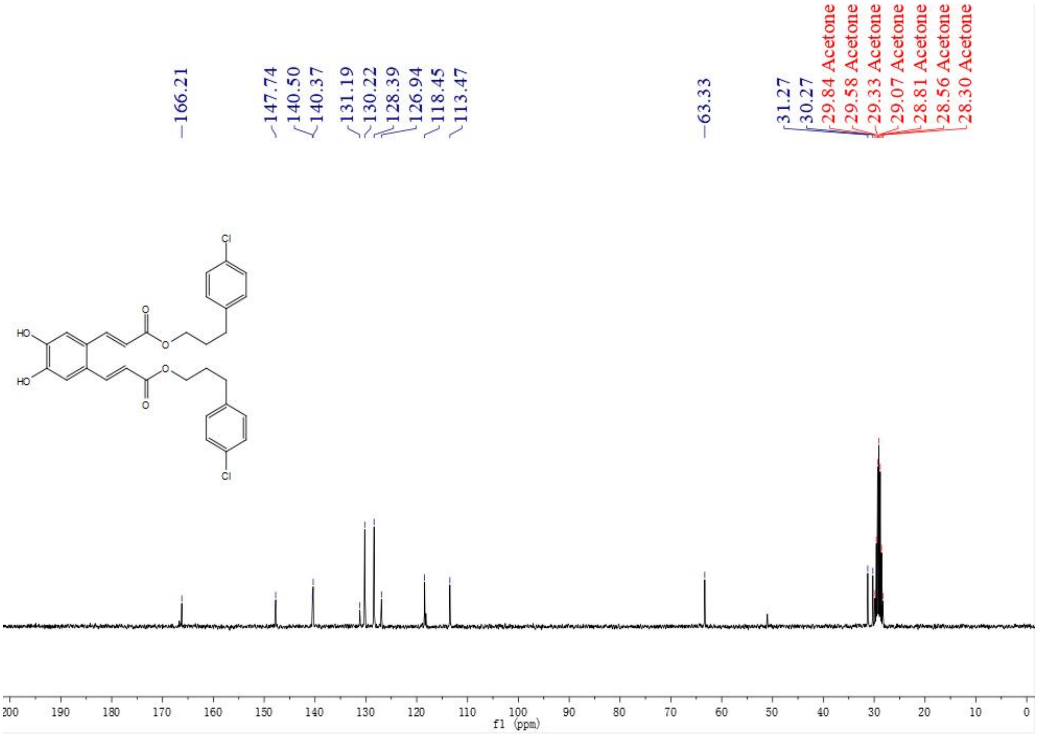

^{1}H NMR Spectrum of **34** (300 MHz, Acetone-d_6)

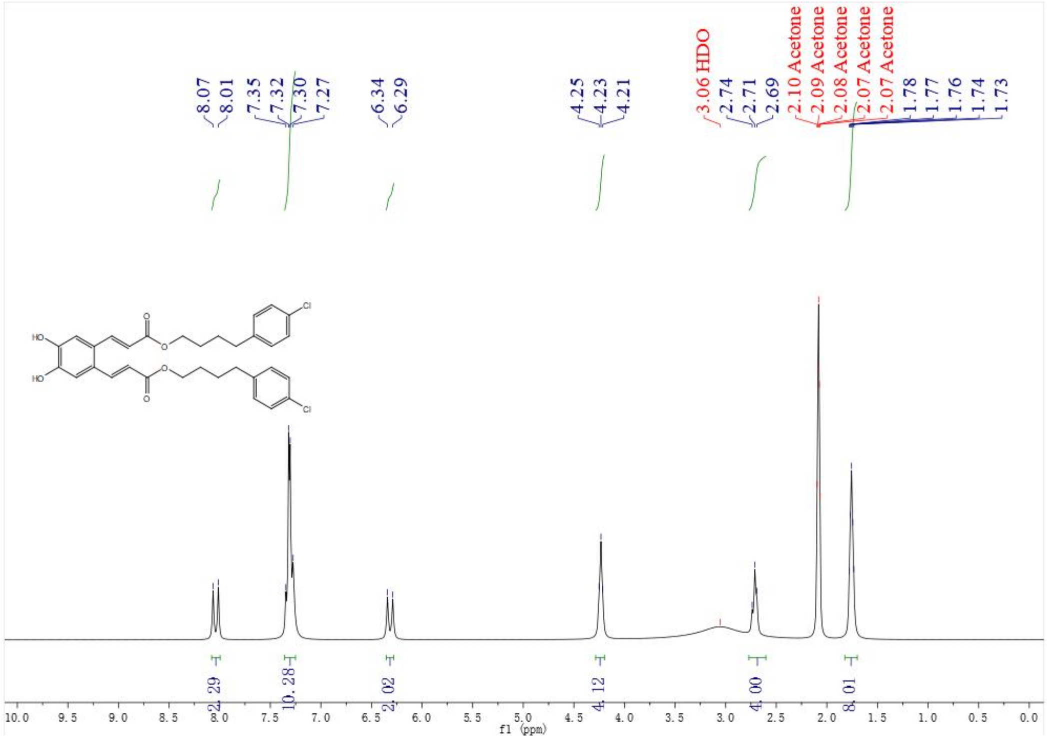

^{13}C NMR Spectrum of **34** (75 MHz, Acetone-d_6)

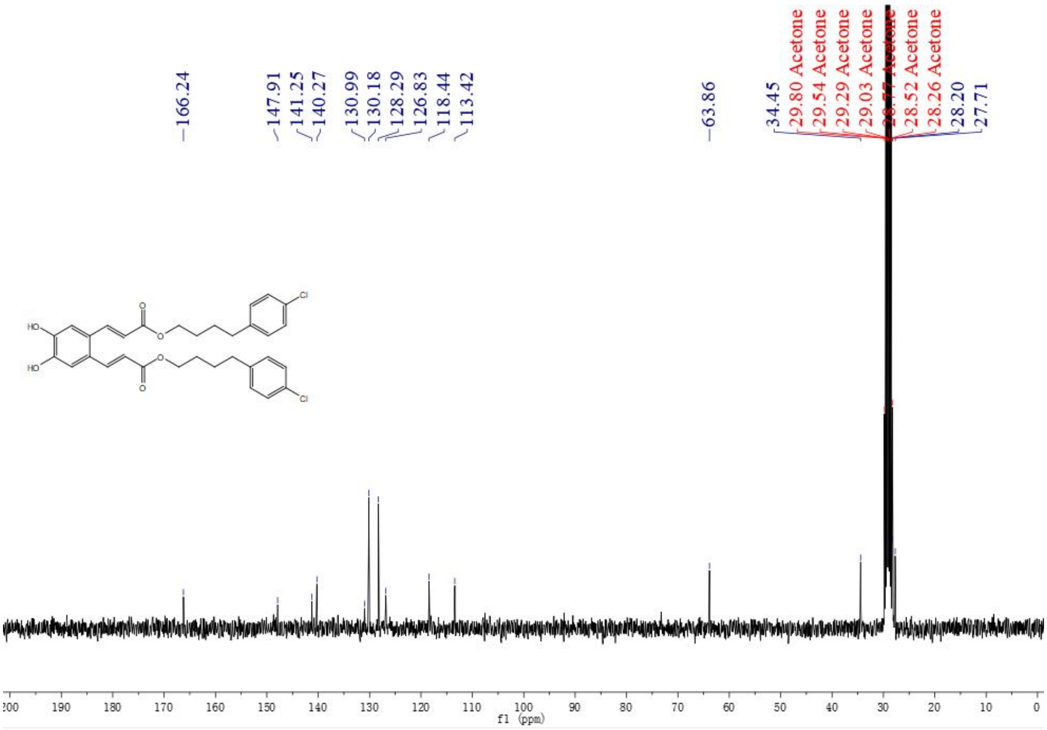

^{1}H NMR Spectrum of **35** (300 MHz, Acetone-d_6)

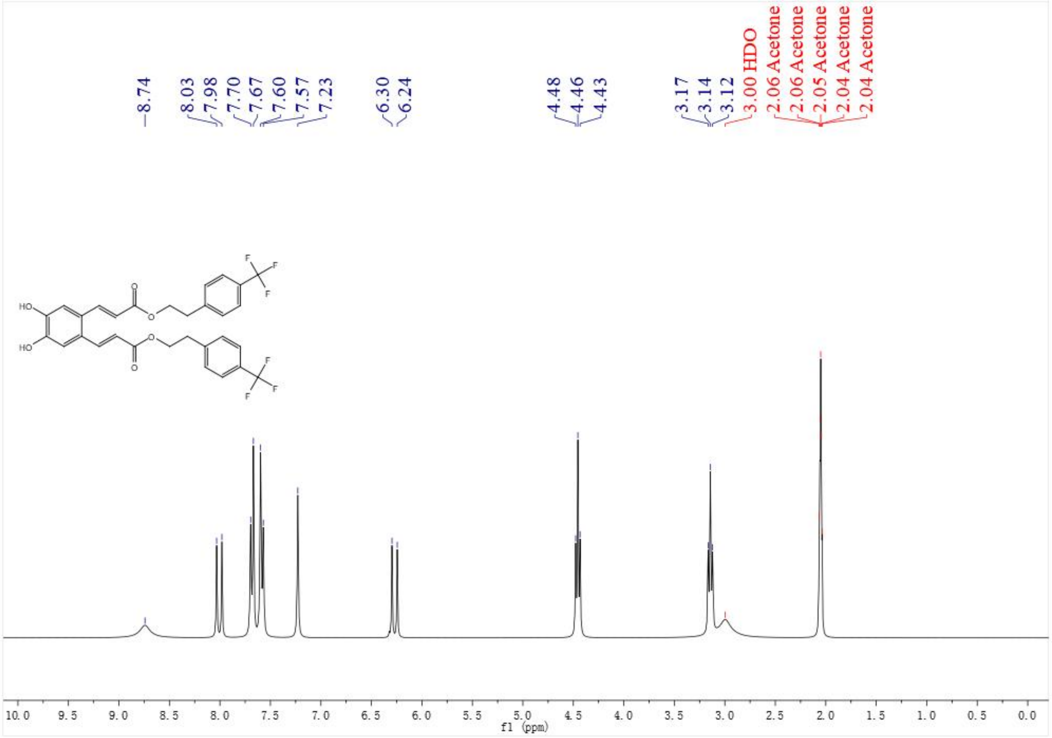

^{13}C NMR Spectrum of **35**(75 MHz, Acetone-d_6)

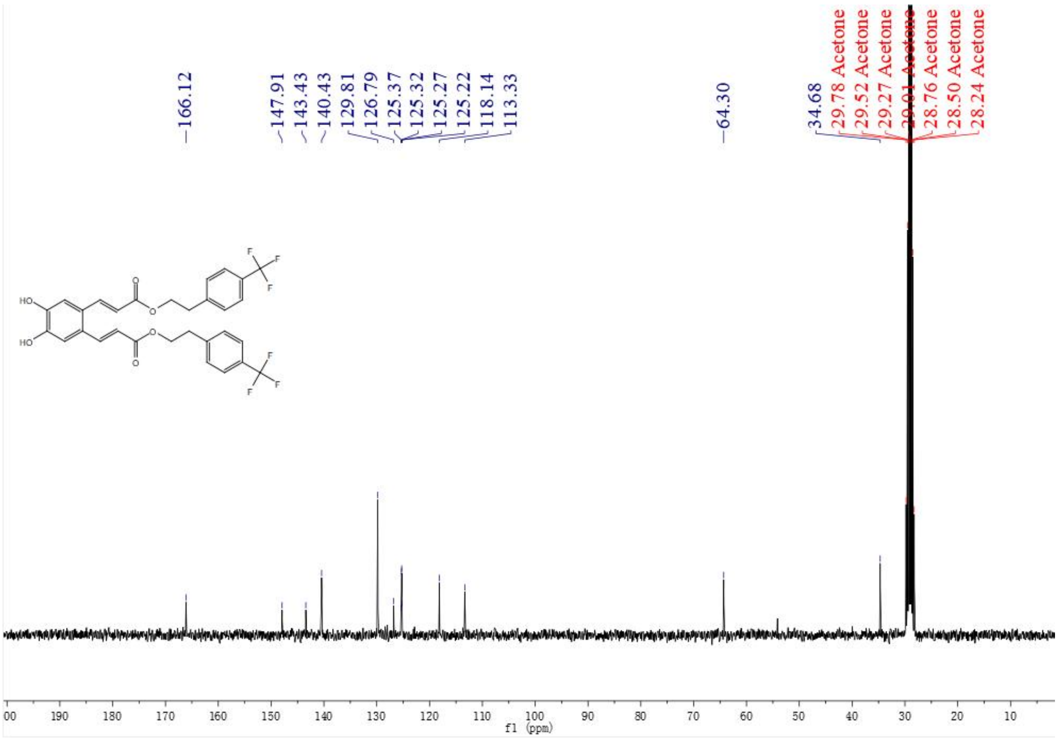

^{1}H NMR Spectrum of **36** (300 MHz, MeOD)

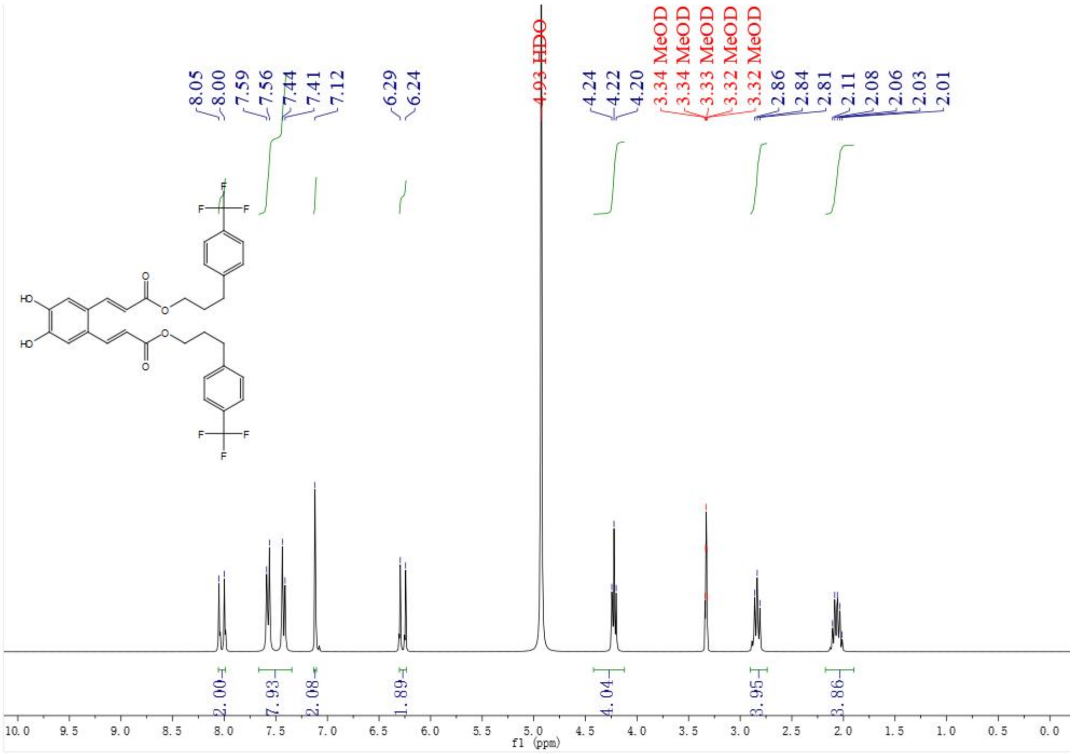

^{13}C NMR Spectrum of **36** (75 MHz, MeOD)

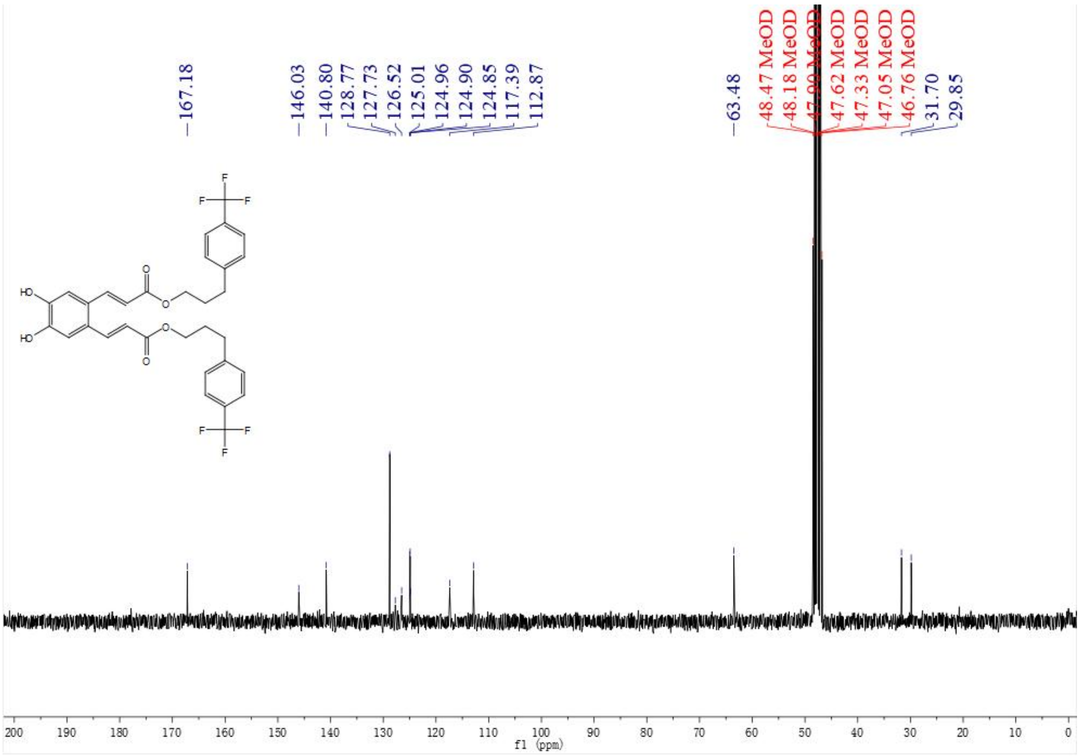

^{1}H NMR Spectrum of **37a** (300 MHz, Acetone-d_6)

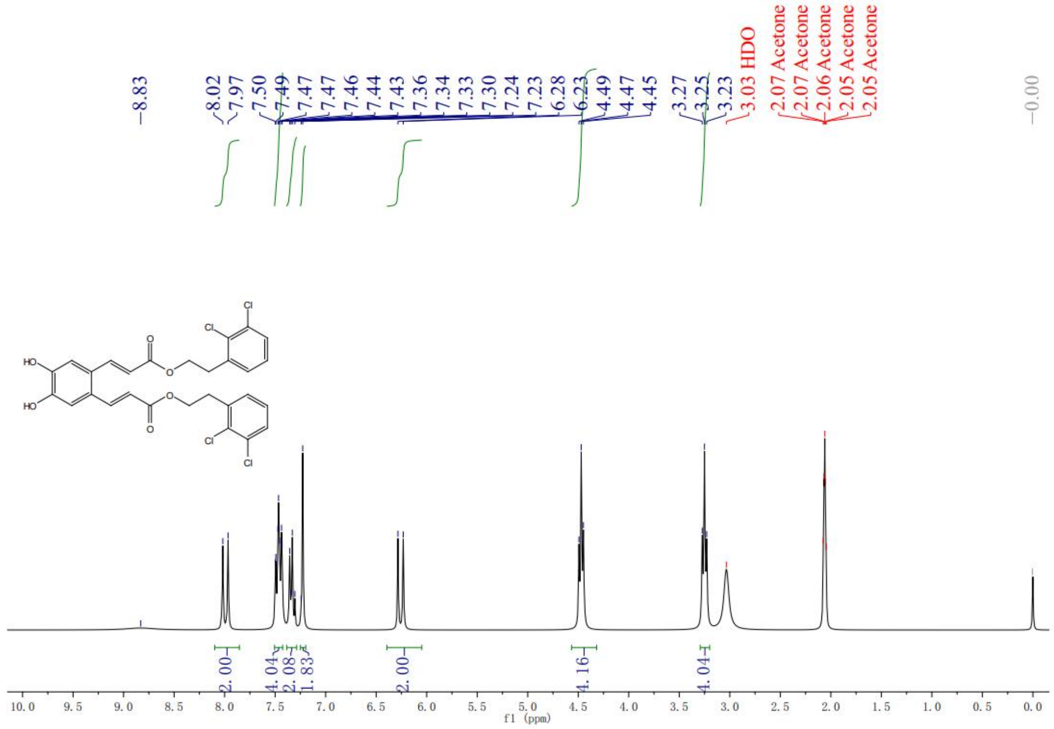

^{13}C NMR Spectrum of **37a** (75 MHz, Acetone-d_6)

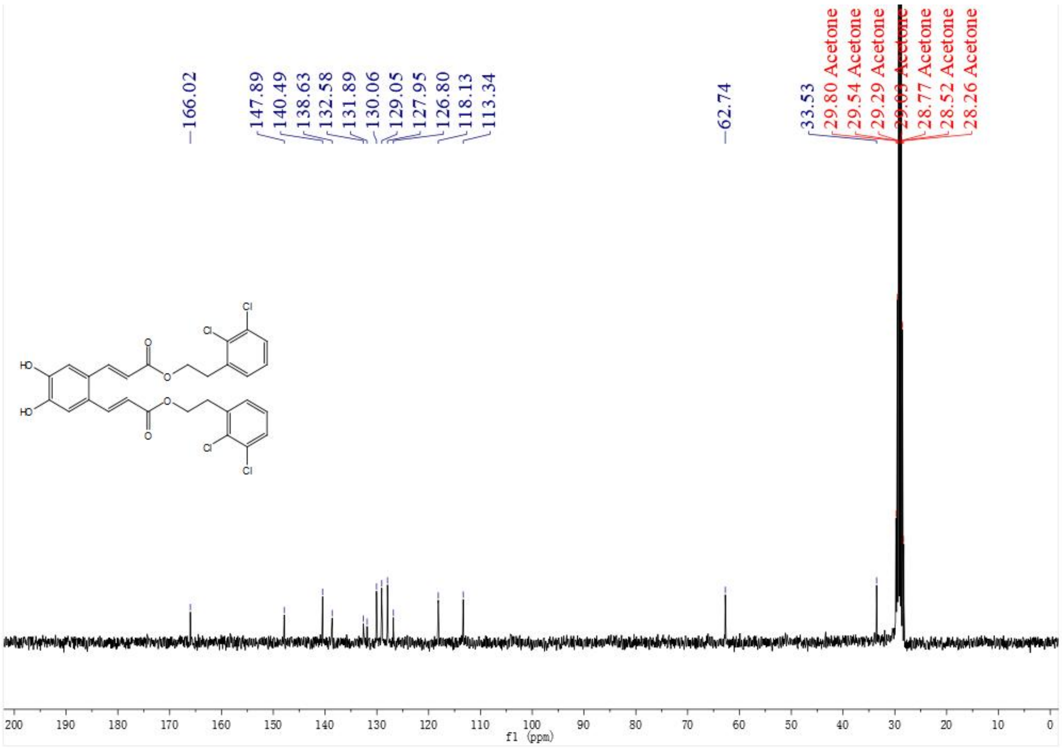

^{1}H NMR Spectrum of **37b** (300 MHz, Acetone-d_6)

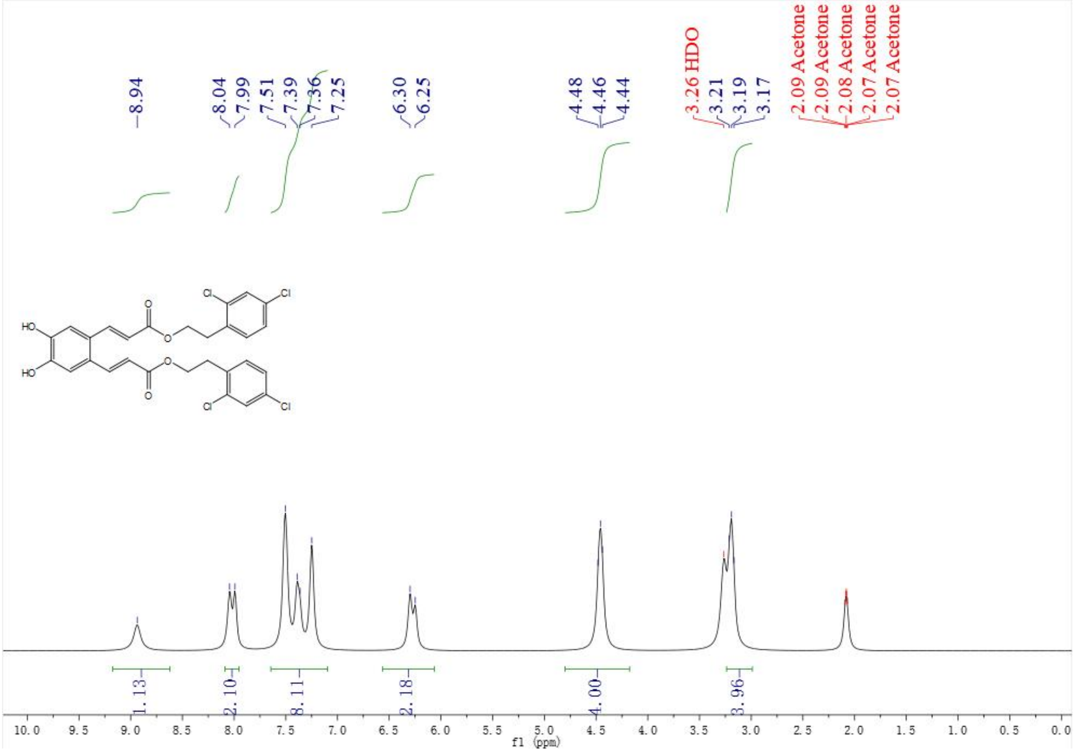

^{13}C NMR Spectrum of **37b** (75 MHz, Acetone-d_6)

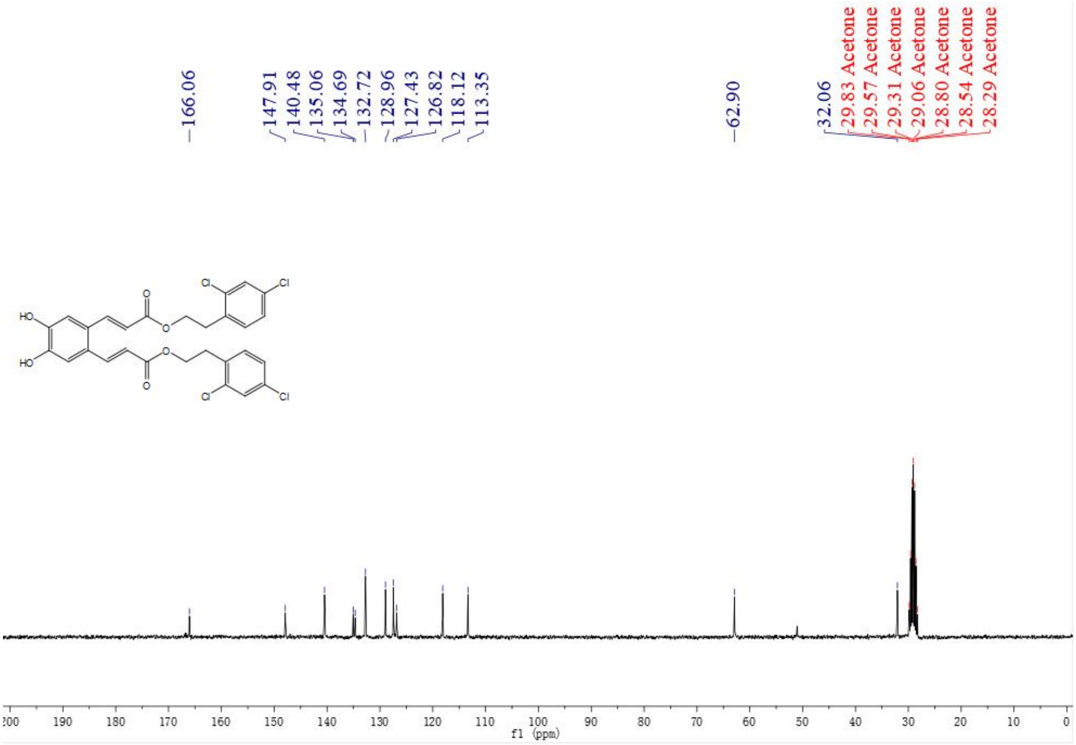

^{1}H NMR Spectrum of **37c** (300 MHz, Acetone-d_6)

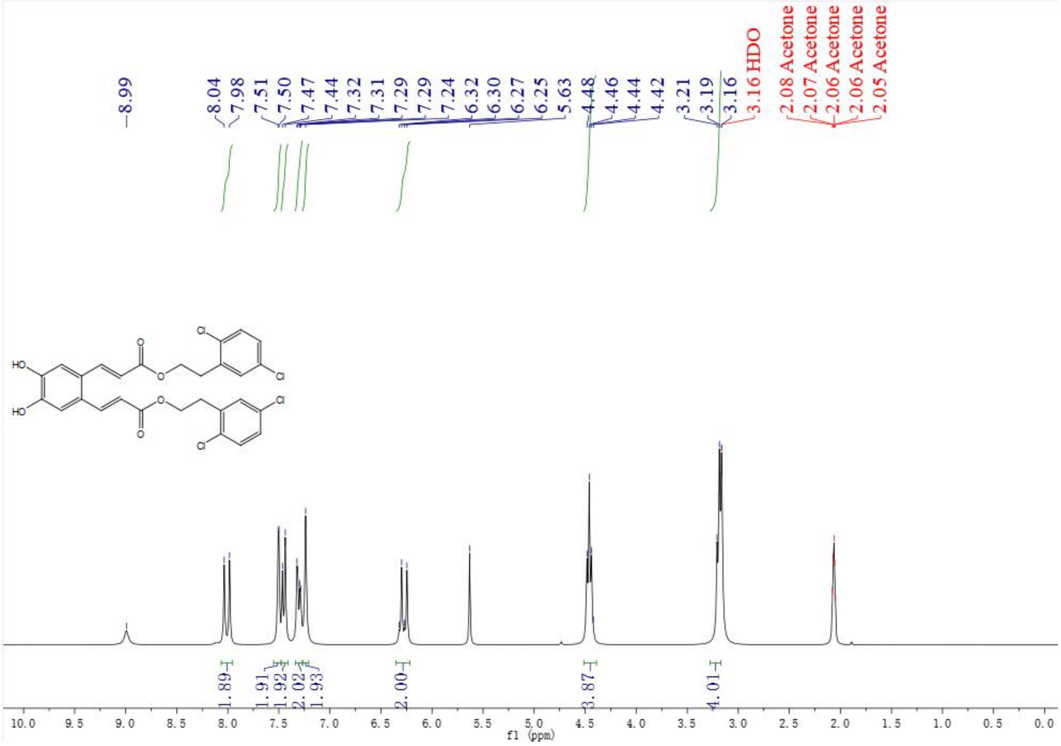

^{13}C NMR Spectrum of **37c** (75 MHz, Acetone-d_6)

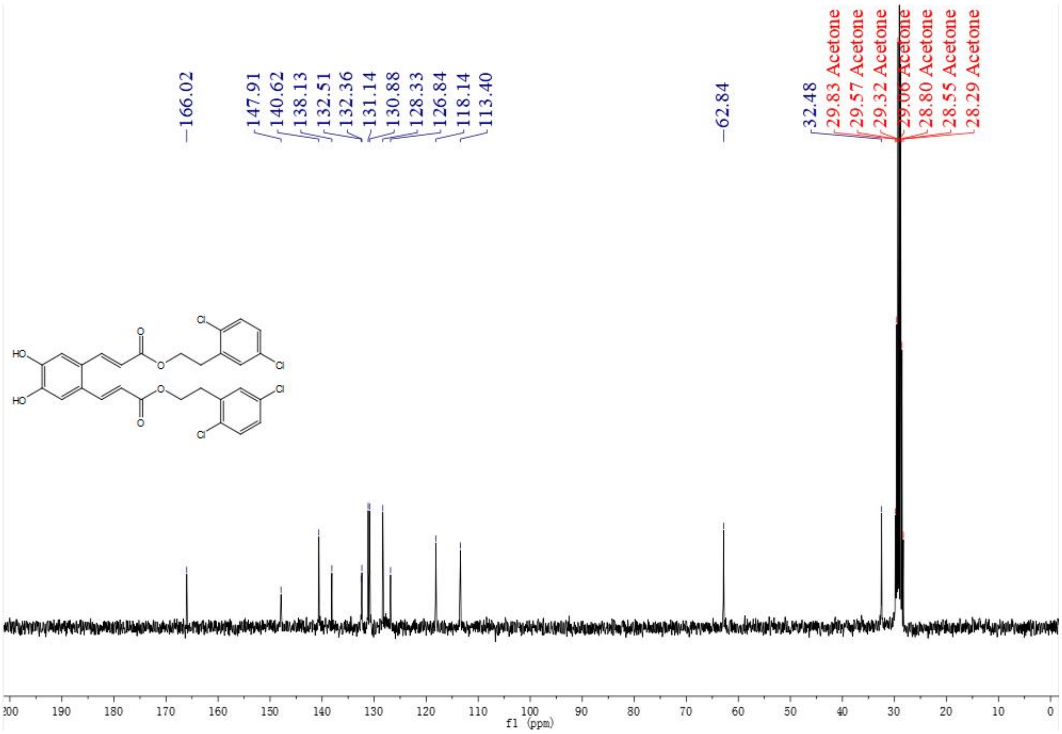

^{1}H NMR Spectrum of **37d** (300 MHz, Acetone-d_6)

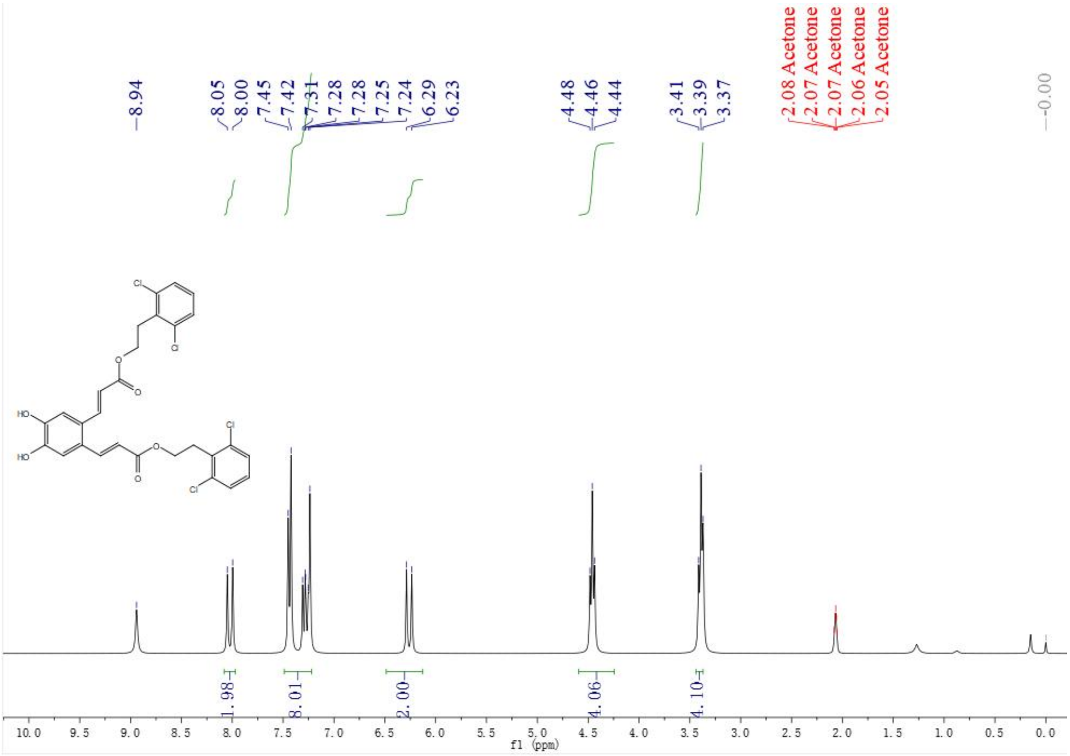

^{13}C NMR Spectrum of **37d** (75 MHz, Acetone-d_6)

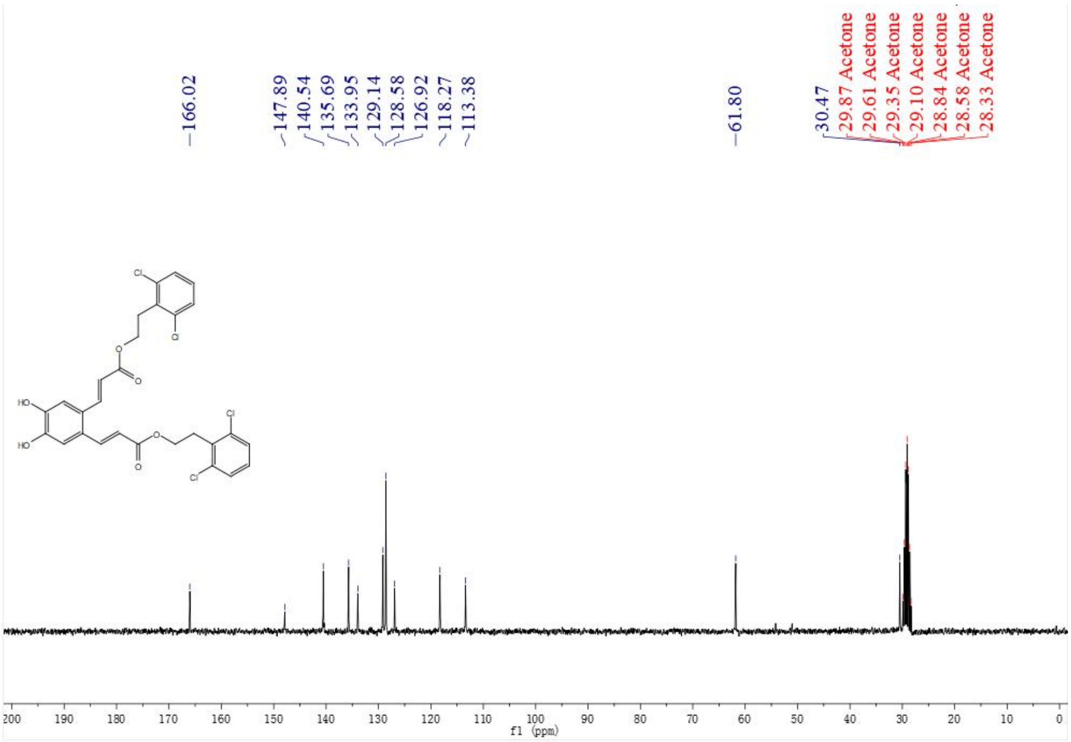

^{1}H NMR Spectrum of **37e** (300 MHz, Acetone-d_6)

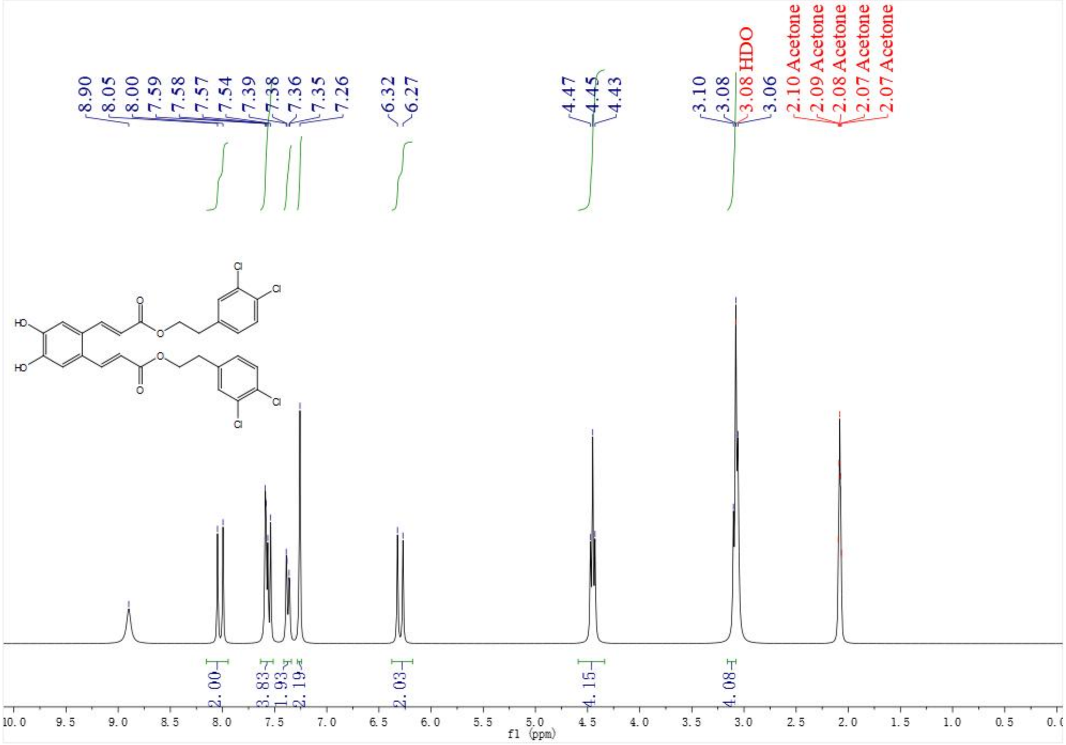

^{13}C NMR Spectrum of **37e** (75 MHz, Acetone-d_6)

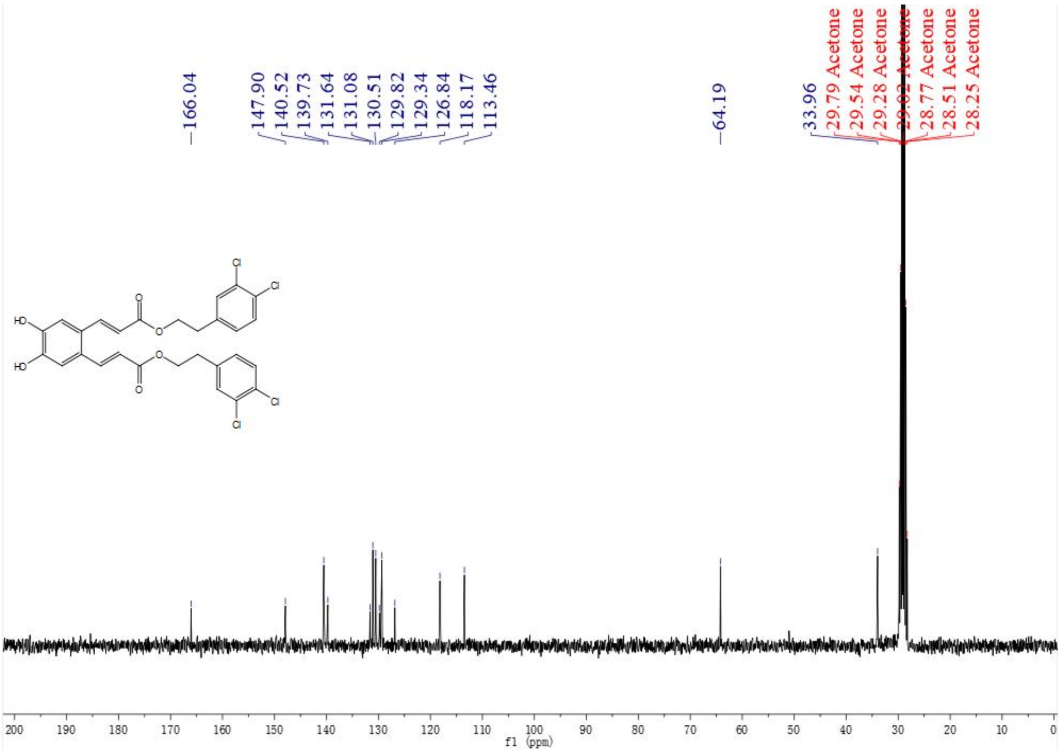

^{1}H NMR Spectrum of **37f** (300 MHz, Acetone-d_6)

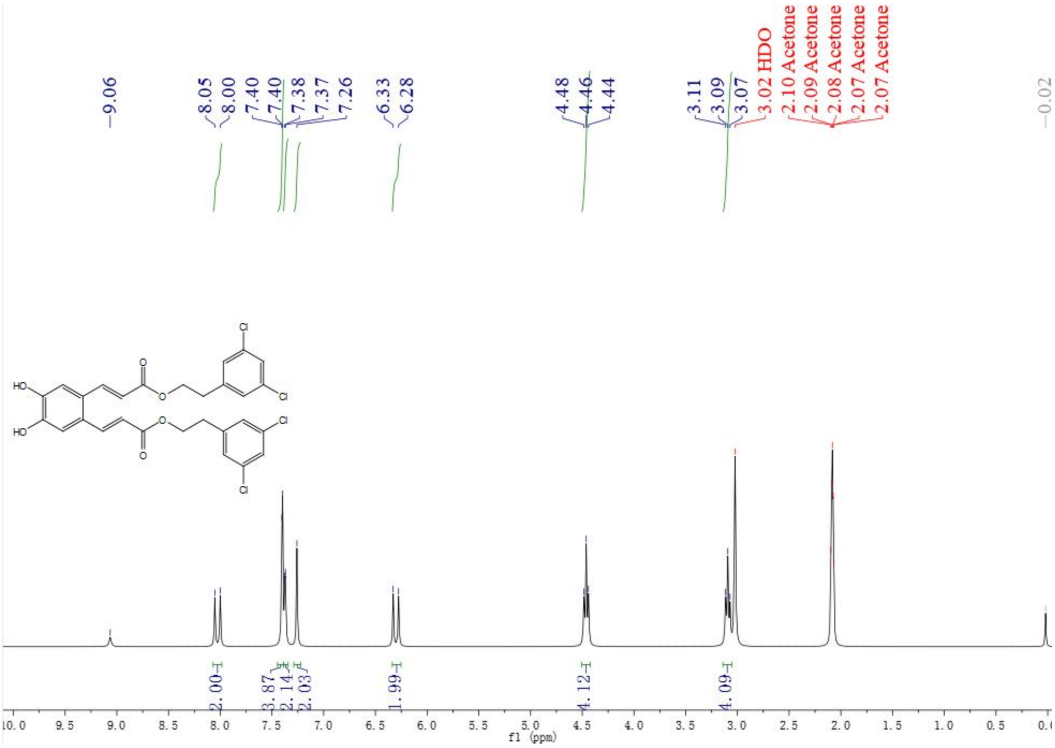

^{13}C NMR Spectrum of **37f** (75 MHz, Acetone-d_6)

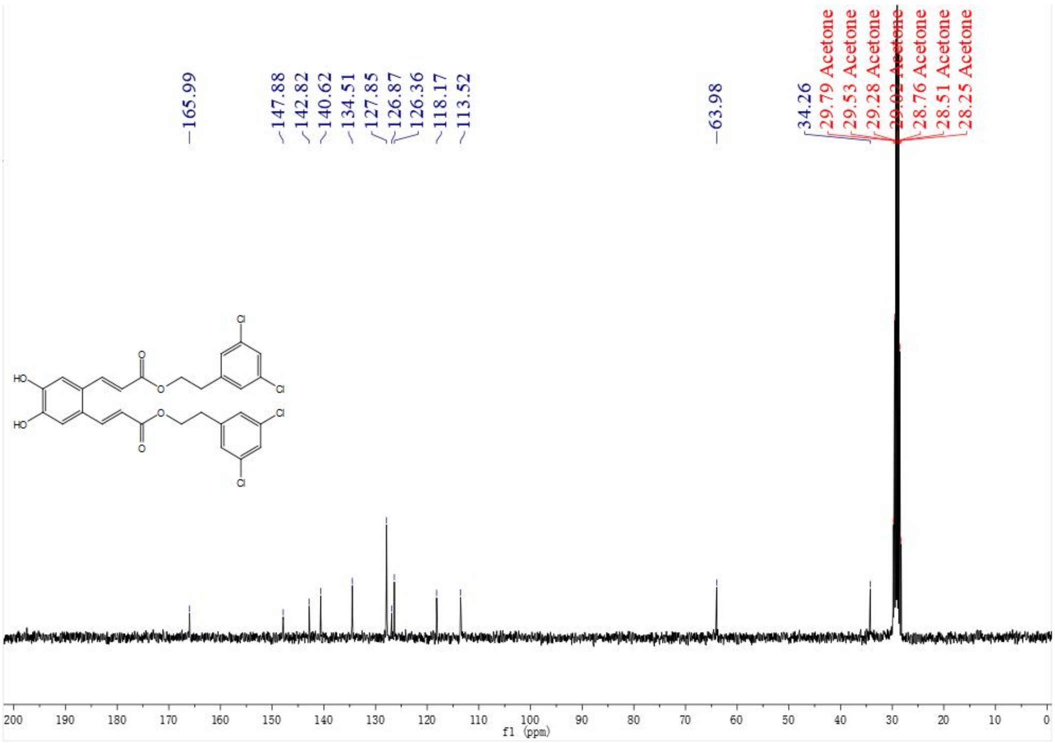

^{1}H NMR Spectrum of **38a** (300 MHz, Acetone-d_6)

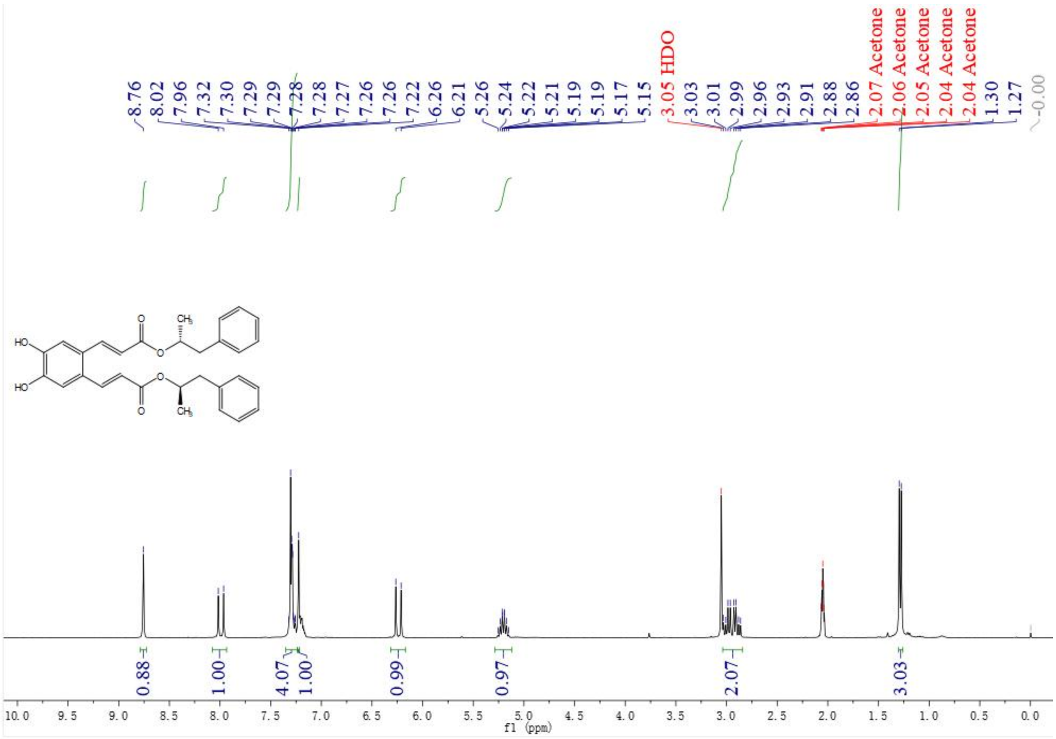

^{13}C NMR Spectrum of **38a** (75 MHz, Acetone-d_6)

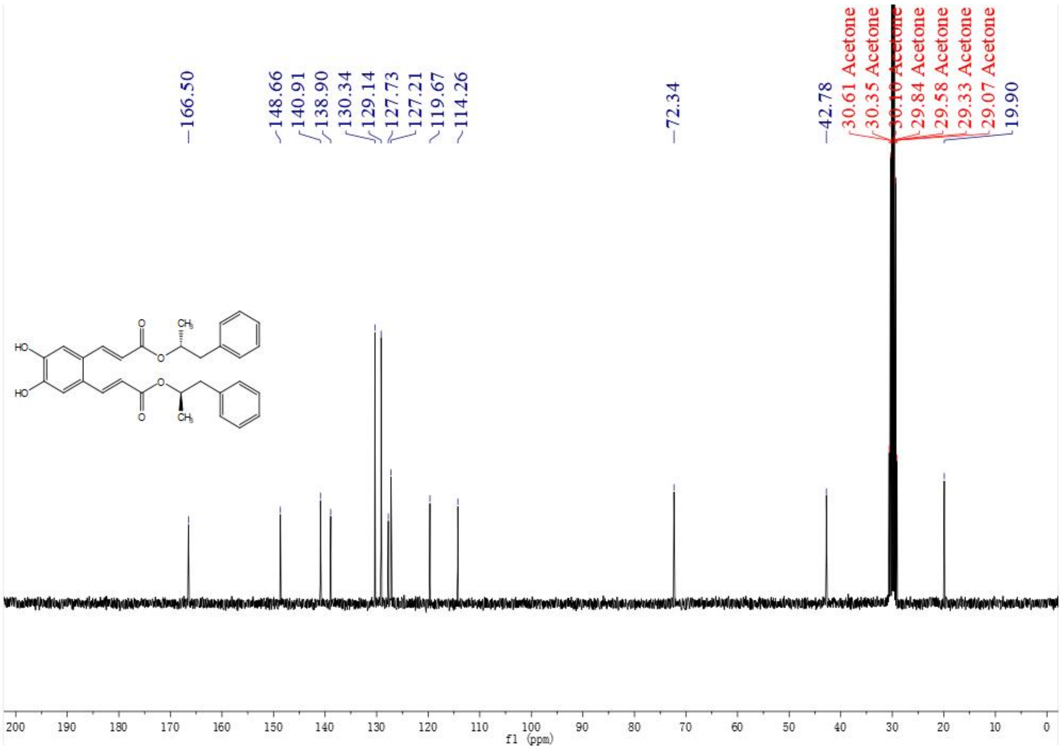

^{1}H NMR Spectrum of **38b** (300 MHz, Acetone-d_6)

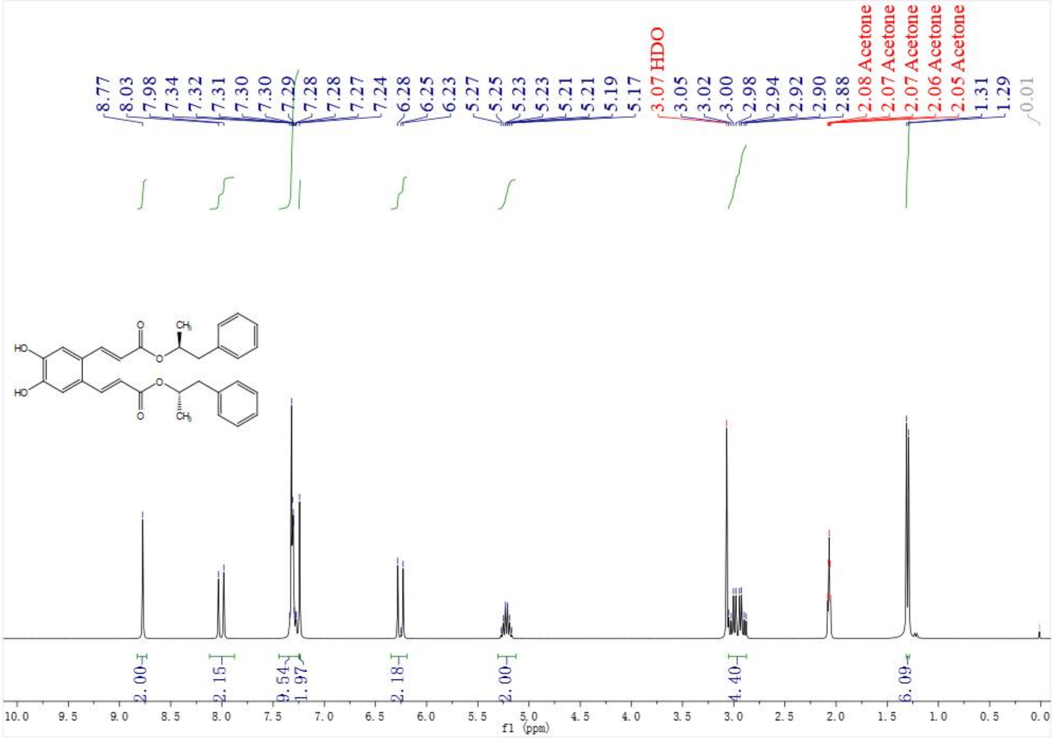

^{13}C NMR Spectrum of **38b** (75 MHz, Acetone-d_6)

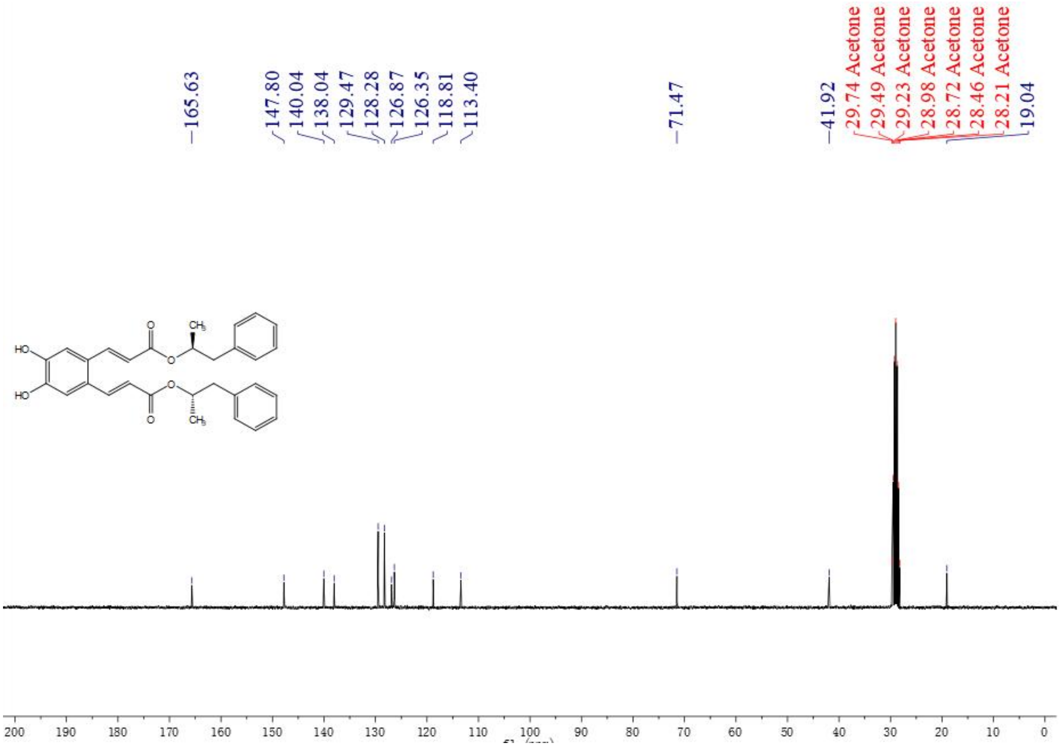

^{1}H NMR Spectrum of **38c** (300 MHz, Acetone-d_6)

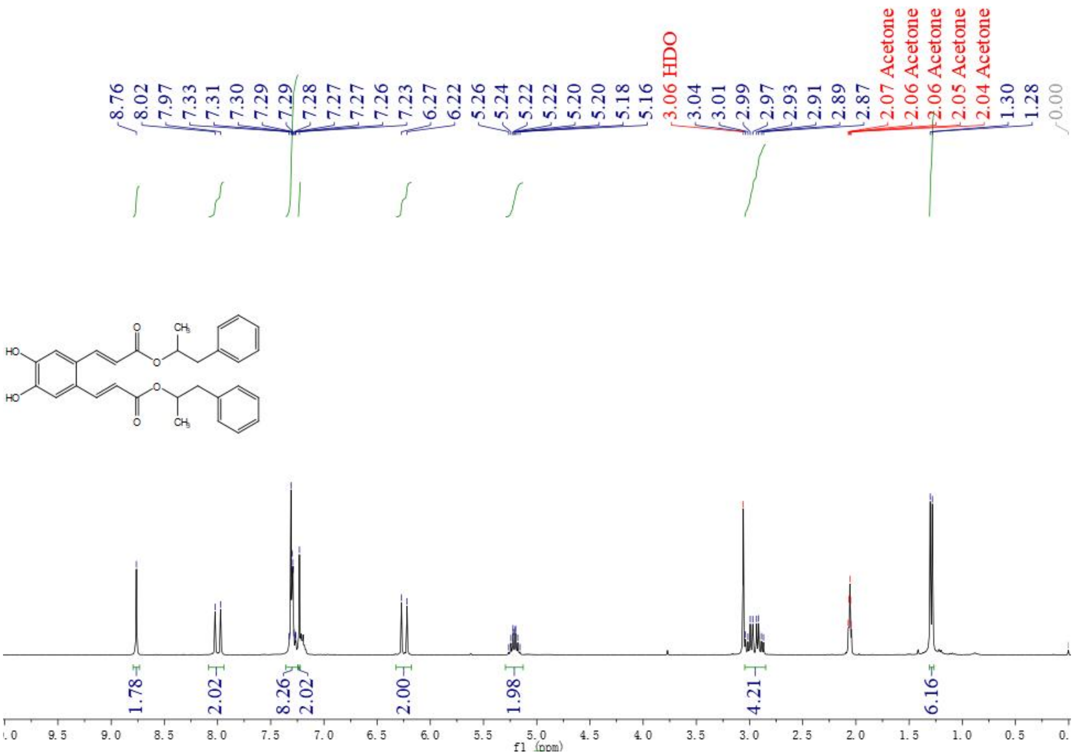

^{13}C NMR Spectrum of **38c** (75 MHz, Acetone-d_6)

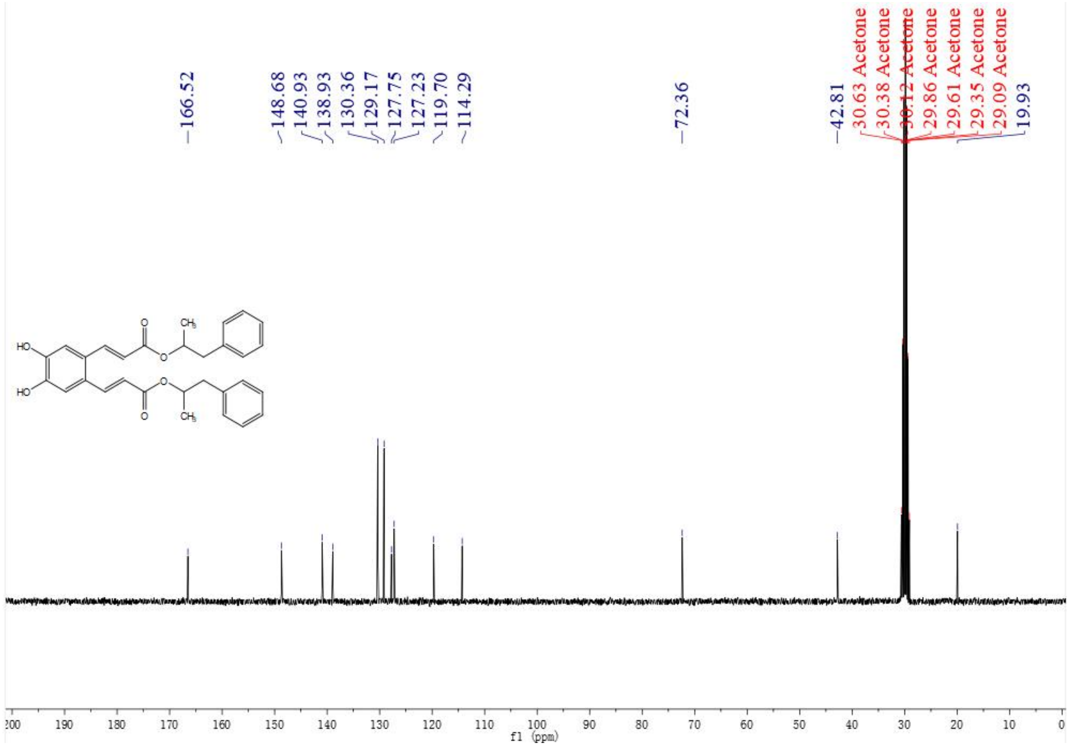

^{1}H NMR Spectrum of **39a** (300 MHz, Acetone-d_6)

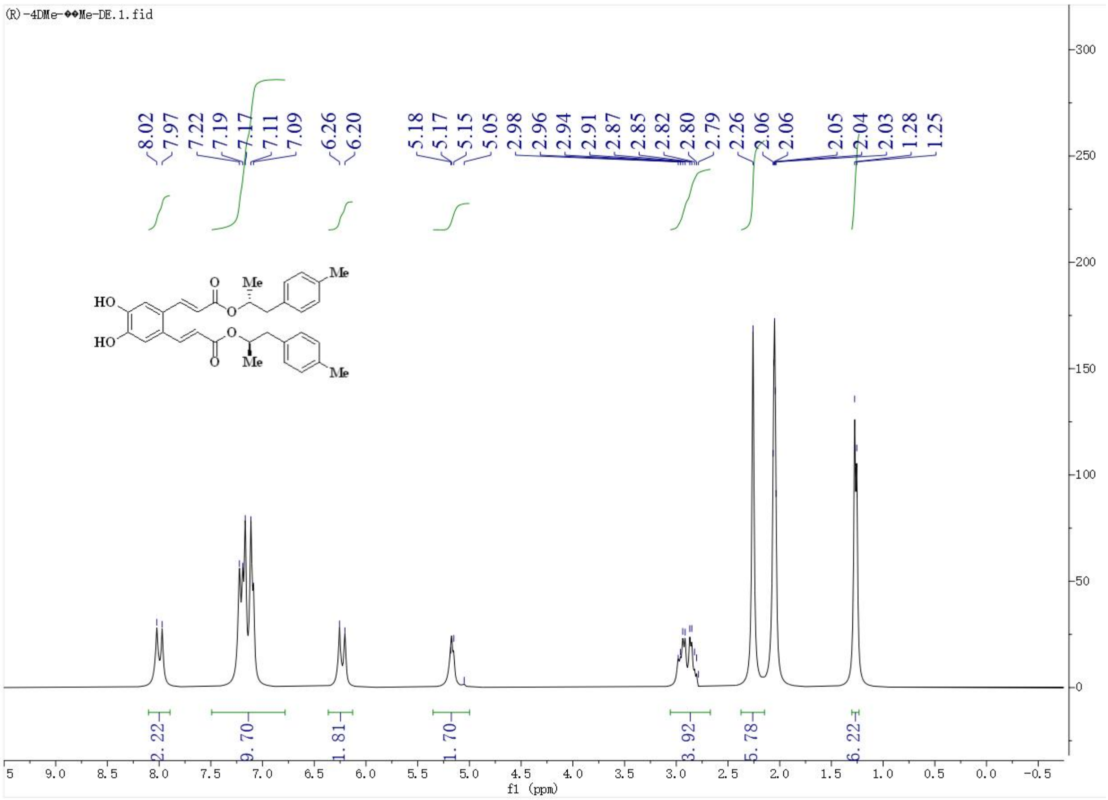

^{13}C NMR Spectrum of **39a** (75 MHz, Acetone-d_6)

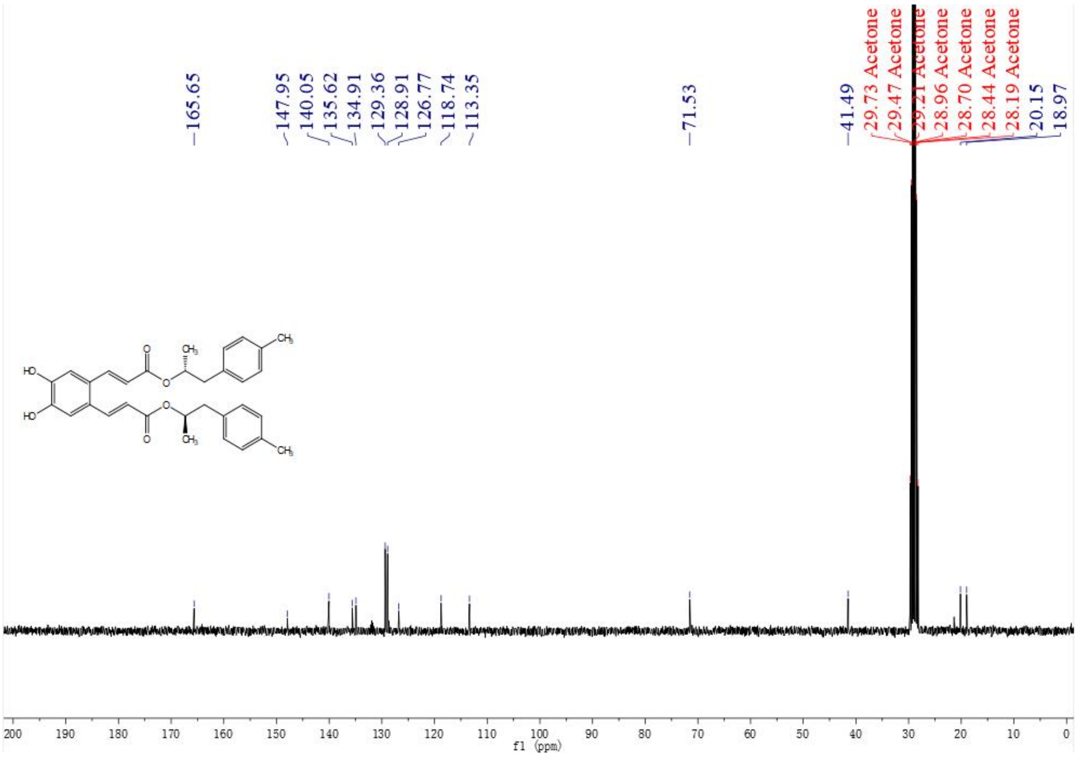

^{1}H NMR Spectrum of **39b** (300 MHz, MeOD)

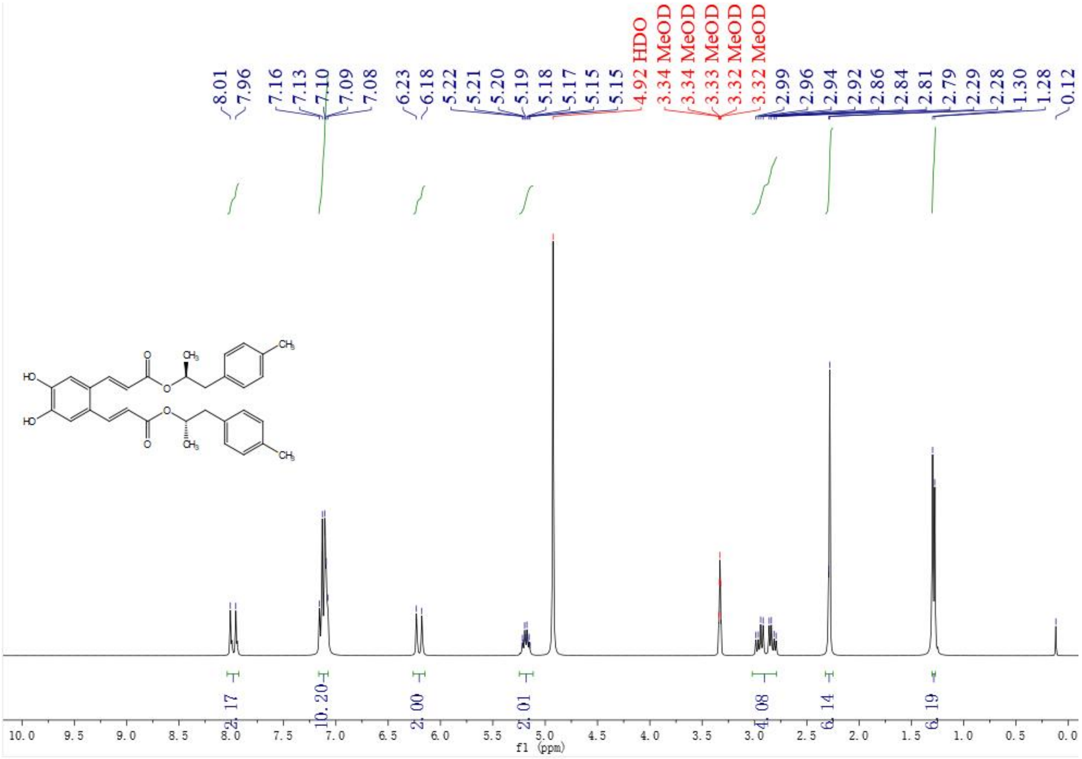

^{13}C NMR Spectrum of **39b** (75 MHz, MeOD)

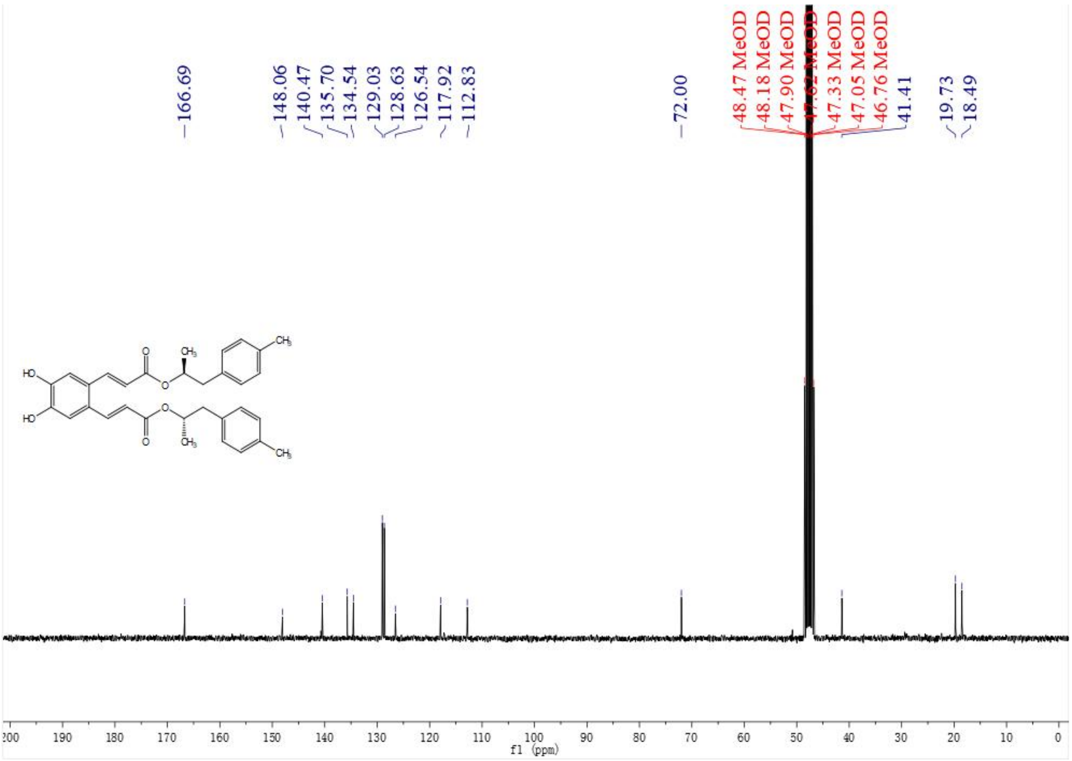

^{1}H NMR Spectrum of **39c** (300 MHz, MeOD)

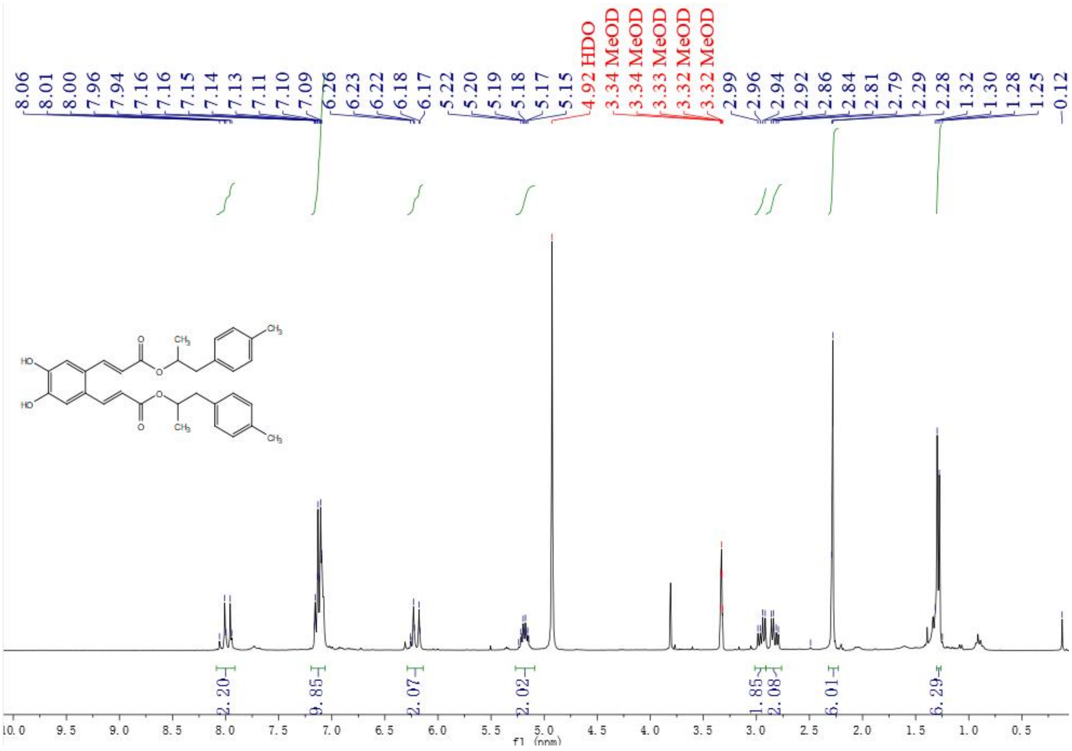

^{13}C NMR Spectrum of **39c** (75 MHz, MeOD)

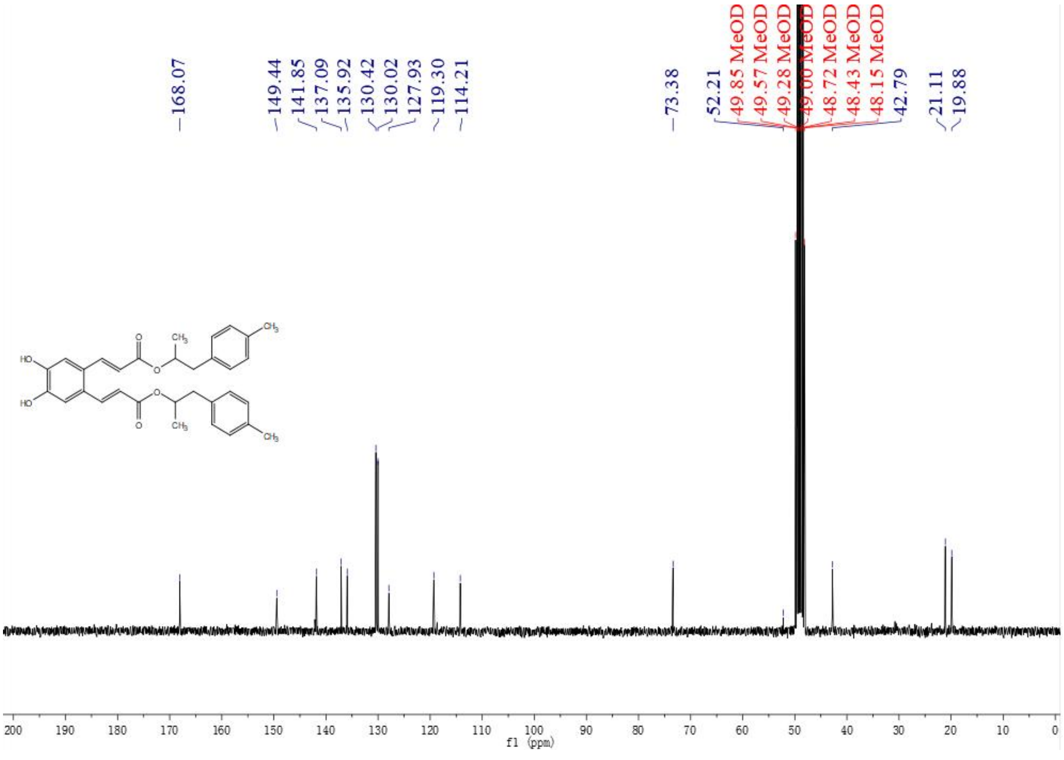

^{1}H NMR Spectrum of **40a** (300 MHz, MeOD)

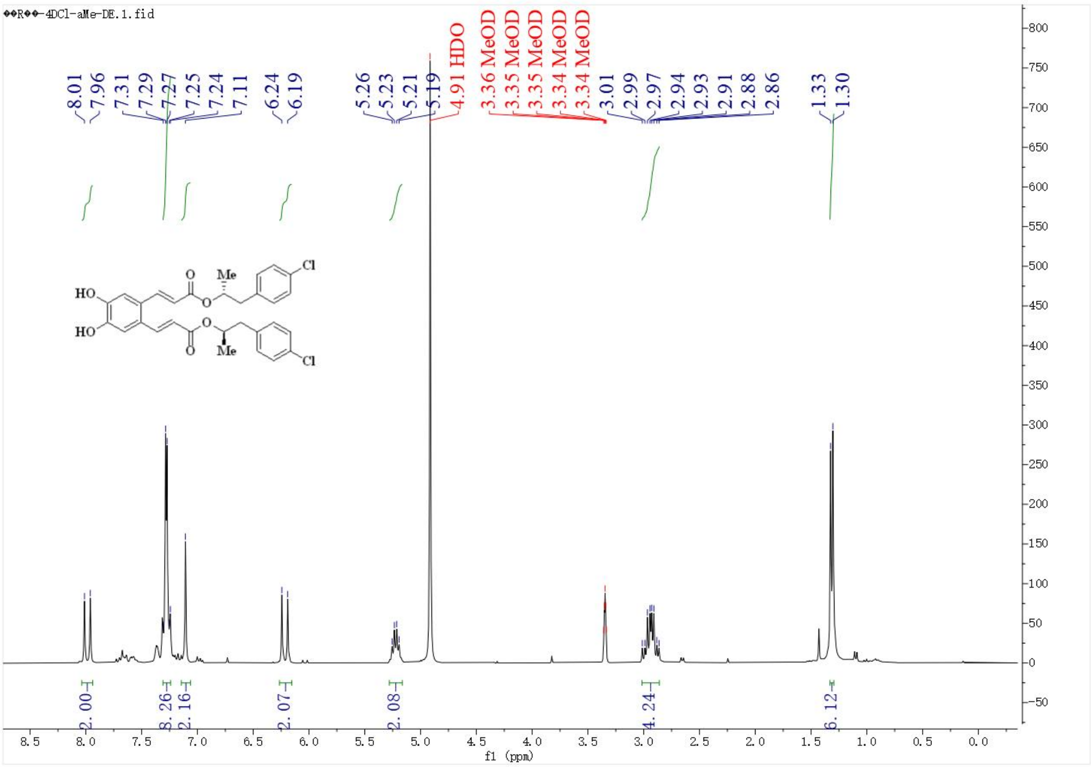

^{13}C NMR Spectrum of **40a** (75 MHz, Acetone-d_6)

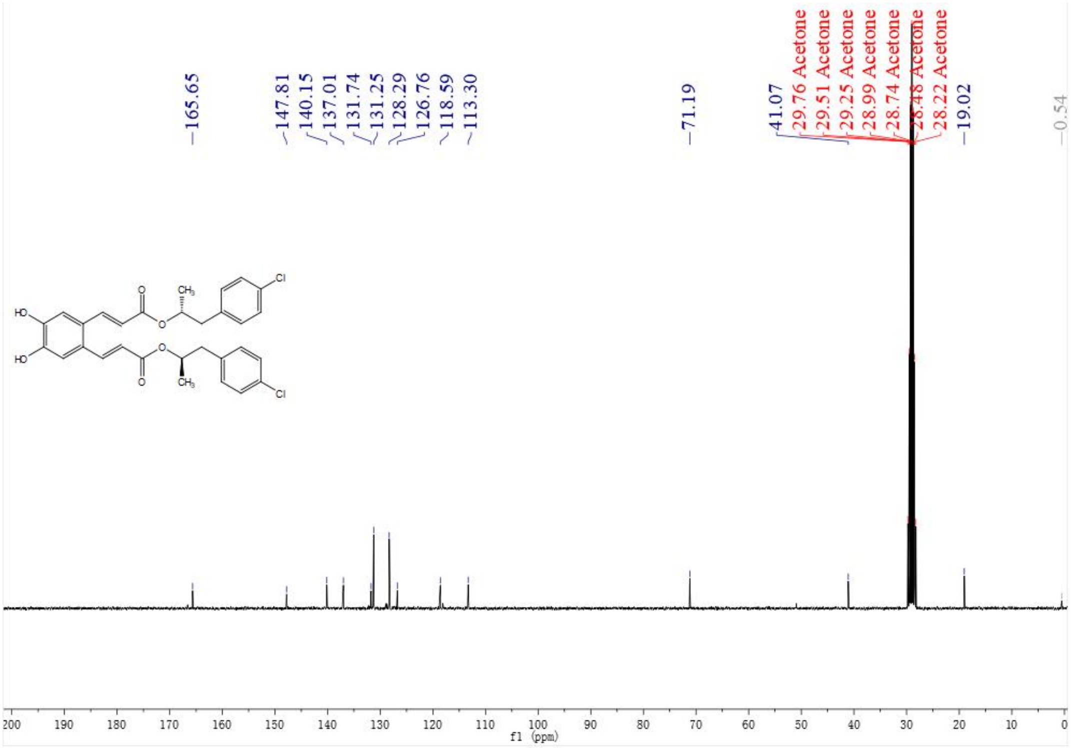

^{1}H NMR Spectrum of **40b** (300 MHz, Acetone-d_6)

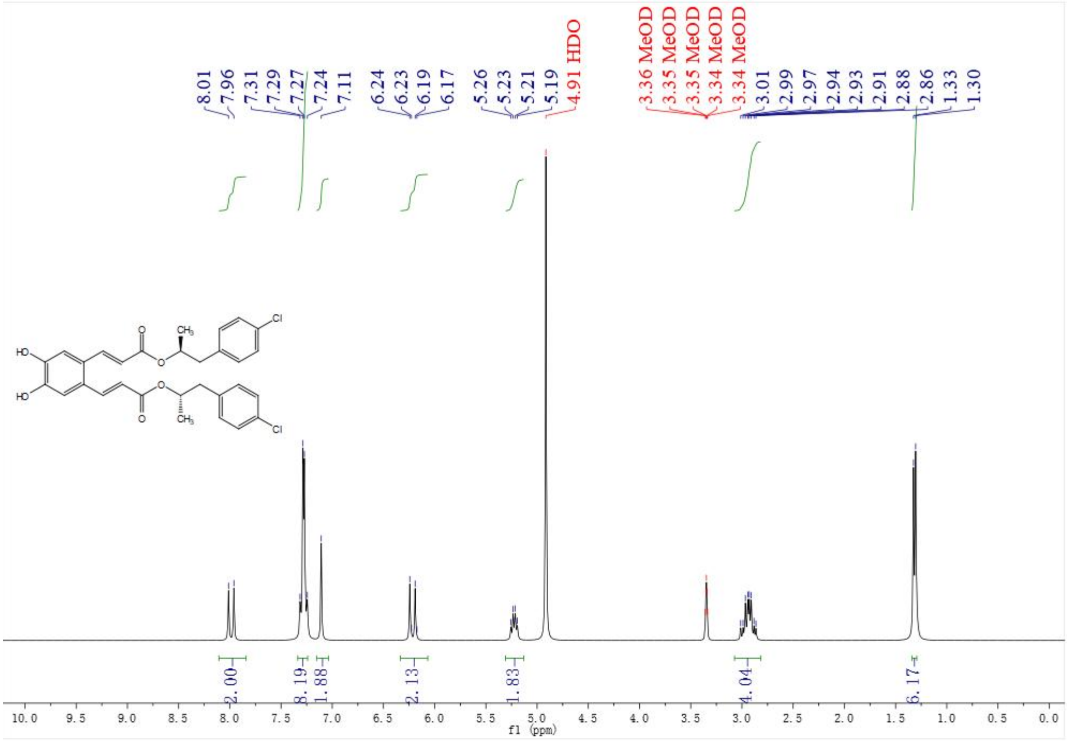

^{13}C NMR Spectrum of **40b** (75 MHz, Acetone-d_6)

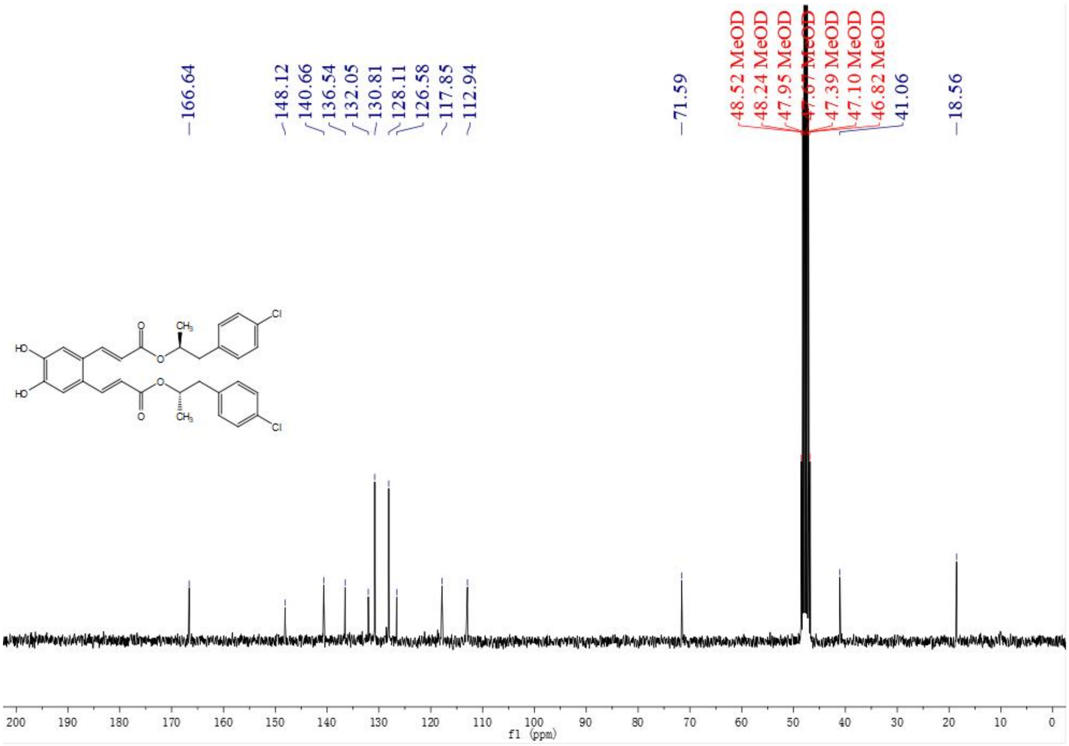

^{1}H NMR Spectrum of **40c** (300 MHz, Acetone-d_6)

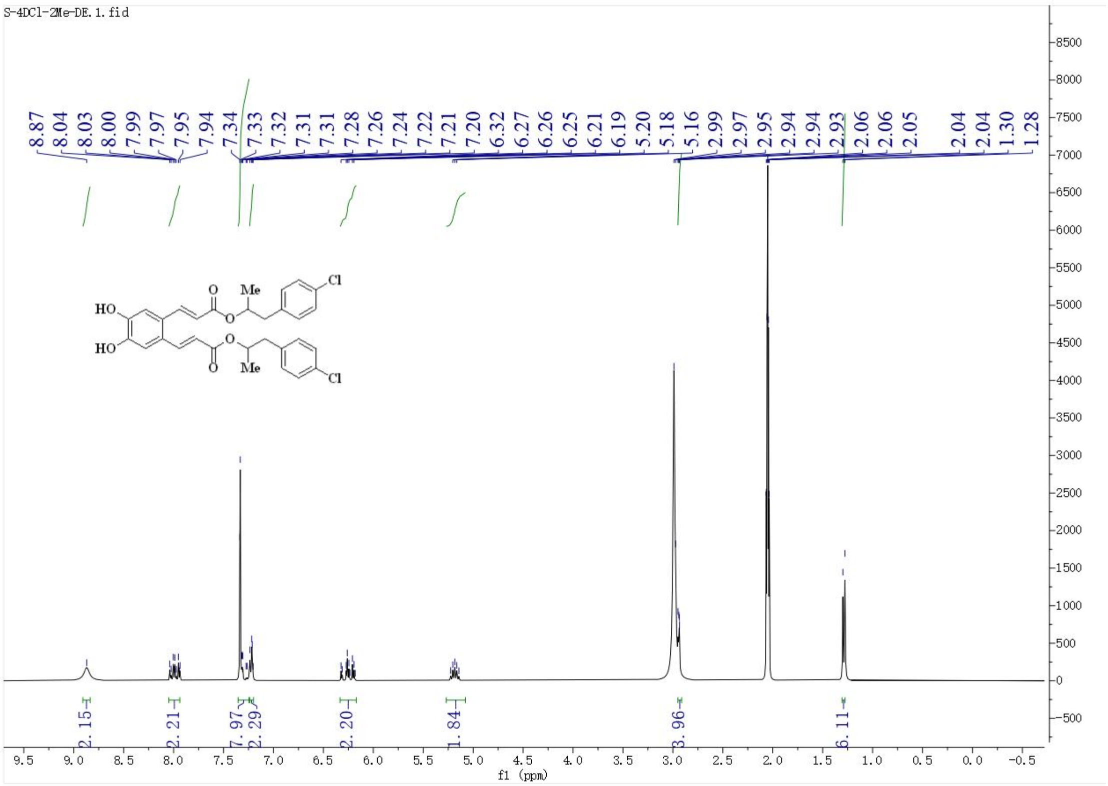

^{13}C NMR Spectrum of **40c** (75 MHz, Acetone-d_6)

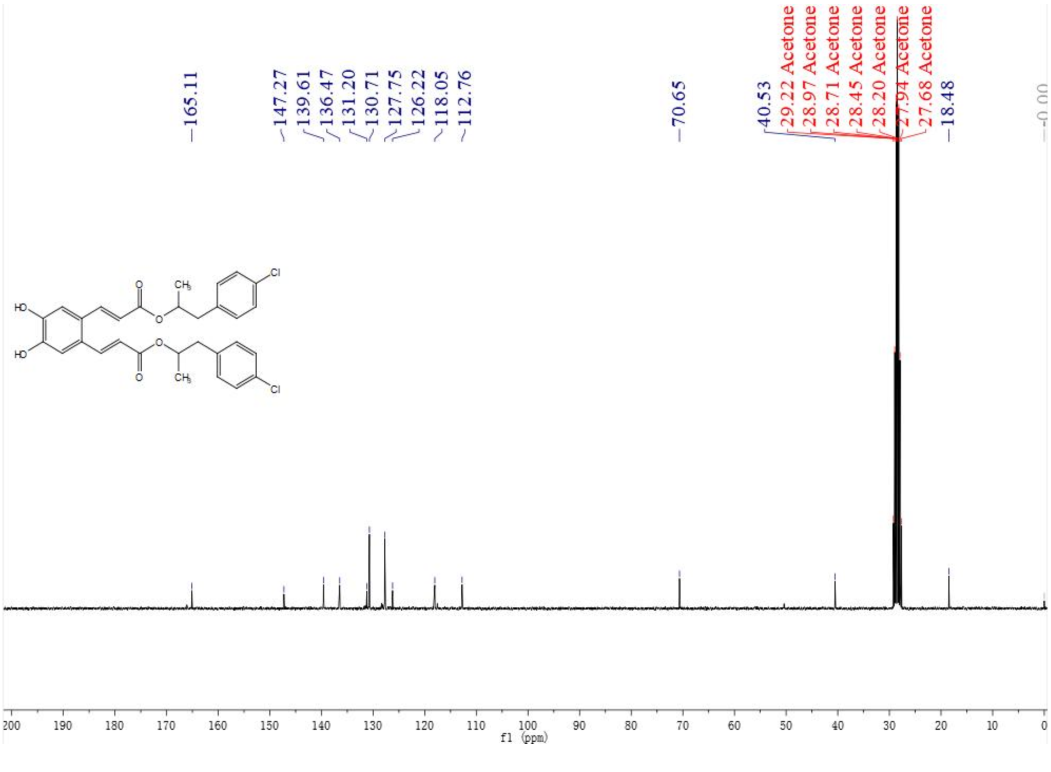

Table S1 Detailed parameters used for molecular docking with DOCK6

Parameters	Data
Name:	WX006.mol
Molecular_Weight:	458.511
DOCK_Rotatable_Bonds:	16
Formal_Charge:	0
HBond_Acceptors:	6
HBond_Donors:	2
Heavy_Atoms:	34
Grid_Score:	-69.330208
Grid_vdw_energy:	-68.342255
Grid_es_energy:	-0.987955
Internal_energy_repulsive:	18.693588

Table S2 Detailed parameters used for molecular docking with Auto Dock Vina

Name	Data
Remark Vina Result	-15.431kJ/mol
Remark Inter+Intra	-15.431kJ/mol
Remark Inter	-15.431kJ/mol
Remark Intra	0
Remark Unbound	0
Remark	0 active torsions:

Table S3 Primer sequences used in the qRT-PCR assay

Name	5' primer	3' primer
SLC7A11	TGCCCTTTCCCTCTATTCGG	TAATGTTCTGGTTATTTTCTCCGAC
HMOX1	AAGACTGCGTTCCTGCTCAA	GGGGGCAGAATCTTGCACT
GSR	TGGGACTCACGGAAGATGAA	ATCGGGGTAAAGCTCGTTGA
GCLC	TGGGGCGATGAGGTGGAATA	GACAGGACCAACCGGACTTT
CHAC1	TCAGTTCCCCCGAAACGAC	ACACGGCCAGGCATCTT
AIFM2	CATGGTGAGGCAGGTCCAG	GGCCACTTGGGAGTGAATGA
FTH1	CCTACGTTTACCTGTCCATGTCTT	GAAGGAAGATTCGGCCACCT
DHODH	GAGACACCTGAAAAAGCGGG	CAGAGTCGGCATCAGGTGTT
ALOX5	CAAAACAGACCCCTGCACAC	CCGGGATTTGGTTGAGCTGG
ACSL4	CCTCCAAGTAGACCAACGCC	TATGTGTCCTTCGGTCCCAG
LPCAT3	TGCGGCTGATCATCTCCATCT	GTGGTAGAGCTGGTTTCCAAAGT
GAPDH	ACCATCTTCCAGGAGCGAGA	GACTCCACGACGTACTCAGC
ATP2A2	TCGAACCCTTGCCACTCATC	CAGGTTCCAGGTAGTTGCGG
CLCC1	GGCATTAGCCATCCTGAGTT	CCTCCTGCCGTCTTCTATCC
ITPR1	CACATTGGACGAGGCTGGAA	ACCTATGACCACGCTGTCTC
SEC61A1	CAACACTGGCCGAGGAATGG	ATTGGCAGGTCCACTCGGA
ATF6	GCAGCACCCAAGACTCAAAC	AGCAAAACCGTCTGGCCTTT
EIF2AK3	AGGGACCTCAAGCCATCCAA	ACTTGTCCTGTGTGTCTGGC
ERN1	TCAATGTCGCTGTGGAGACC	ACATACAGAGTGGGCGTCAG
